# Targeting Zfp36 to combat cardiac hypertrophy: Insights into ferroptosis pathways

**DOI:** 10.1002/ctm2.70247

**Published:** 2025-02-25

**Authors:** Mingyu Zhang, Xiaoxiang Guan, Zheng Dong, Chenxu Yang, Chao Xiong, Wenzheng Cheng, Aijing Shang, Yaru Liu, Xiaofei Guo, Bowen Zhang, Bo Zhang, Saidi Jin, Wenyi Qi, Berezhnova Tatjana Alexandrovna, Yuan Jiang, Zhimin Du, Chaoqian Xu

**Affiliations:** ^1^ State Key Laboratory of Frigid Zone Cardiovascular Diseases (SKLFZCD) Department of Pharmacology (State Key Labratoray ‐Province Key Laboratories of Biomedicine‐Pharmaceutics of China Key Laboratory of Cardiovascular Research, Ministry of Education) College of Pharmacy Harbin Medical University Harbin China; ^2^ Department of Clinical Pharmacy the First Affiliated Hospital of Harbin Medical University Harbin China; ^3^ Department of Pharmacology, Voronezh State Medical University Named After N.N. Burdenko Voronezh; ^4^ Department of Biopharmaceutical Sciences, College of Pharmacy Harbin Medical University Harbin China; ^5^ State Key Laboratory of Quality Research in Chinese Medicines Macau University of Science and Technology Macau China; ^6^ Joint International Research Laboratory of Cardiovascular Medicine Research Ministry of Education Harbin China

**Keywords:** cardiac hypertrophy, ferroptosis, RNA binding, Ythdc2, Zfp36

## Abstract

**Background:**

Cardiac hypertrophy is a precursor to heart failure and represents a significant global cause of mortality, thereby necessitating timely and effective therapeutic interventions. Zinc finger protein 36 (Zfp36) is recognised as a critical regulator of ferroptosis; however, its role and underlying mechanisms in cardiac hypertrophy remain largely unexplored. This study aims to investigate the regulatory function of Zfp36 in ferroptosis within the context of cardiac hypertrophy.

**Methods and results:**

Single‐cell sequencing analysis demonstrated a reduction in Zfp36 expression associated with cardiac hypertrophy. Zfp36 was observed to mitigate ferroptosis and reduce hypertrophic phenotypes in cardiomyocytes subjected to Angiotensin II (Ang II) and in myocardial tissues induced by transverse aortic constriction. The ferroptosis inhibitor Ferrostatin‐1 was shown to alleviate hypertrophy when co‐incubated with si‐Zfp36 and Ang II. Mechanistically, Zfp36 binds to the 3′ untranslated region (3′UTR) of Ythdc2 mRNA, facilitating its degradation. Ythdc2 subsequently binds to SLC7A11 mRNA, enhancing its decay, which leads to a reduction in glutathione (GSH) levels, thereby exacerbating ferroptosis and cardiac hypertrophy. Furthermore, overexpression of Ythdc2 reversed the protective effects conferred by Zfp36, while silencing of Ythdc2 counteracted the effects of Zfp36 knockdown.

**Conclusions:**

This study elucidates the role of Zfp36 in cardiac hypertrophy, specifically detailing its modulatory mechanism via the Ythdc2/SLC7A11/GSH ferroptosis pathway. These insights lay the groundwork for innovative approaches to understanding the pathological mechanisms underlying cardiac hypertrophy and enhancing clinical interventions.

**Key points:**

- Zfp36 was initially demonstrated to attenuate cardiac hypertrophy through the inhibition of ferroptosis in cardiomyocytes, providing a new target for therapeutic strategies targeting ferroptosis.
- Zfp36 facilitated the degradation of Ythdc2 mRNA by binding to it, subsequently inhibiting Ythdc2‐mediated degradation of SLC7A11 mRNA, and maintaining GSH levels. This elucidates a previously unrecognized regulatory pathway in the context of cardiac hypertrophy.

## Graphical abstract

Zinc finger protein 36 bounds to the 3′ untranslated region of Ythdc2 mRNA, facilitating its degradation. Ythdc2 can bind to SLC7A11 mRNA to accelerate its decay, thereby reducing glutathione levels and aggravating ferroptosis‐induced cardiac hypertrophy.

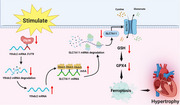

## 1 INTRODUCTION

Cardiac hypertrophy is usually marked by a rise in heart mass in response to pathological factors such as hypertension and stenosis of the aortic or mitral valves. This response progresses through adaptive and maladaptive stages, ultimately culminating in heart failure, which significantly and independently raises the risk of cardiovascular disease morbidity and mortality.^1^, ^2^ Maladaptive decompensation in cardiac hypertrophy, alongside cell growth and protein synthesis, occurs in the presence of cell death, fibrosis, mitochondrial dysfunction, reactivation of foetal gene expression, and in adequate angiogenesis.^3^ Recent studies have highlighted the role of programmed cell death mechanisms, including ferroptosis, pyroptosis, necroptosis and cuproptosis, in the regulation of various cardiovascular diseases.^4^, ^5^, ^6^ Notably, ferroptosis has been closely associated with the onset and progression of pathological cardiac hypertrophy.^7^

Ferroptosis, an emerging form of cell death, is characterised as an iron‐dependent form of programmed cell death, involving the accumulation of lipid peroxides, iron overload, and the diminished antioxidative capacity within the System X_c_^−^/glutathione (GSH) peroxidase 4 (Gpx4) signalling pathway.^8^ In the last 10 years, ferroptosis has become an important factor in the development and advancement of several cardiovascular diseases, such as drug‐induced heart failure, myocardial ischaemia reperfusion injury and sepsis‐induced cardiomyopathy.^9^ Recent studies show that ferroptosis is essential in the development of cardiac hypertrophy, indicating that addressing ferroptosis might offer substantial therapeutic opportunities to alleviate cardiac hypertrophy.^10^, ^11^, ^12^ Further studies are necessary to understand the mechanisms through which ferroptosis influences the pathogenesis of hypertrophic cardiomyopathy.

Zfp36 is an RNA‐binding protein (RBP) that plays a crucial role in the destabilisation of mRNAs containing AU‐rich elements (AREs).^13^ The tandem CCCH zinc fingers are used to bind to AREs, after which it recruits the mRNA decapping factor DCP2, the CCR4‐NOT deadenylase complex, and the RNA‐degrading exosome, facilitating the breakdown of target mRNAs.^14^ This mechanism positions Zfp36 as a potential therapeutic target for various human diseases, including rheumatoid arthritis, psoriasis, obesity and pulmonary inflammation.^14^, ^15^ Recent research indicates that Zfp36 plays a role in the regulation of ferroptosis, suggesting that Zfp36‐dependent ferroptosis could serve as a potential therapeutic target for viral myocarditis.^16^ An integrated analysis has identified zfp36 as a key target in dilated cardiomyopathy.^17^ Specifically, Zfp36 in vascular smooth muscle cells regulates vessel contractility.^18^ Furthermore, Zfp36 has been found to mitigate the inflammatory response in aortic endothelial cells and modulate atherosclerosis.^19^ Collectively, these findings underscore the involvement of Zfp36 in cardiovascular diseases; however, its role and underlying molecular mechanisms in relation to ferroptosis in cardiac hypertrophy remain unexplored.

In this study, we observed a reduction in Zfp36 levels during cardiac hypertrophy. Cardiac‐specific overexpression of Zfp36 mitigated cardiac hypertrophy by inhibiting ferroptosis, whereas Zfp36 deficiency aggravated cardiac hypertrophy by promoting ferroptosis. Additionally, we identified Ythdc2 as a target of Zfp36. This interaction contributed to cardiac hypertrophy by binding to SLC7A11 mRNA, promoting its degradation, and consequently accelerating ferroptosis through the inhibition of GSH synthesis. The findings of this study collectively indicate that targeting Zfp36 may represent a viable therapeutic strategy to prevent the transition from cardiac hypertrophy to heart failure.

## 2 RESULTS

### 2.1 Ferroptosis‐associated Zfp36 expression is reduced under cardiac hypertrophy

**FIGURE 1:**
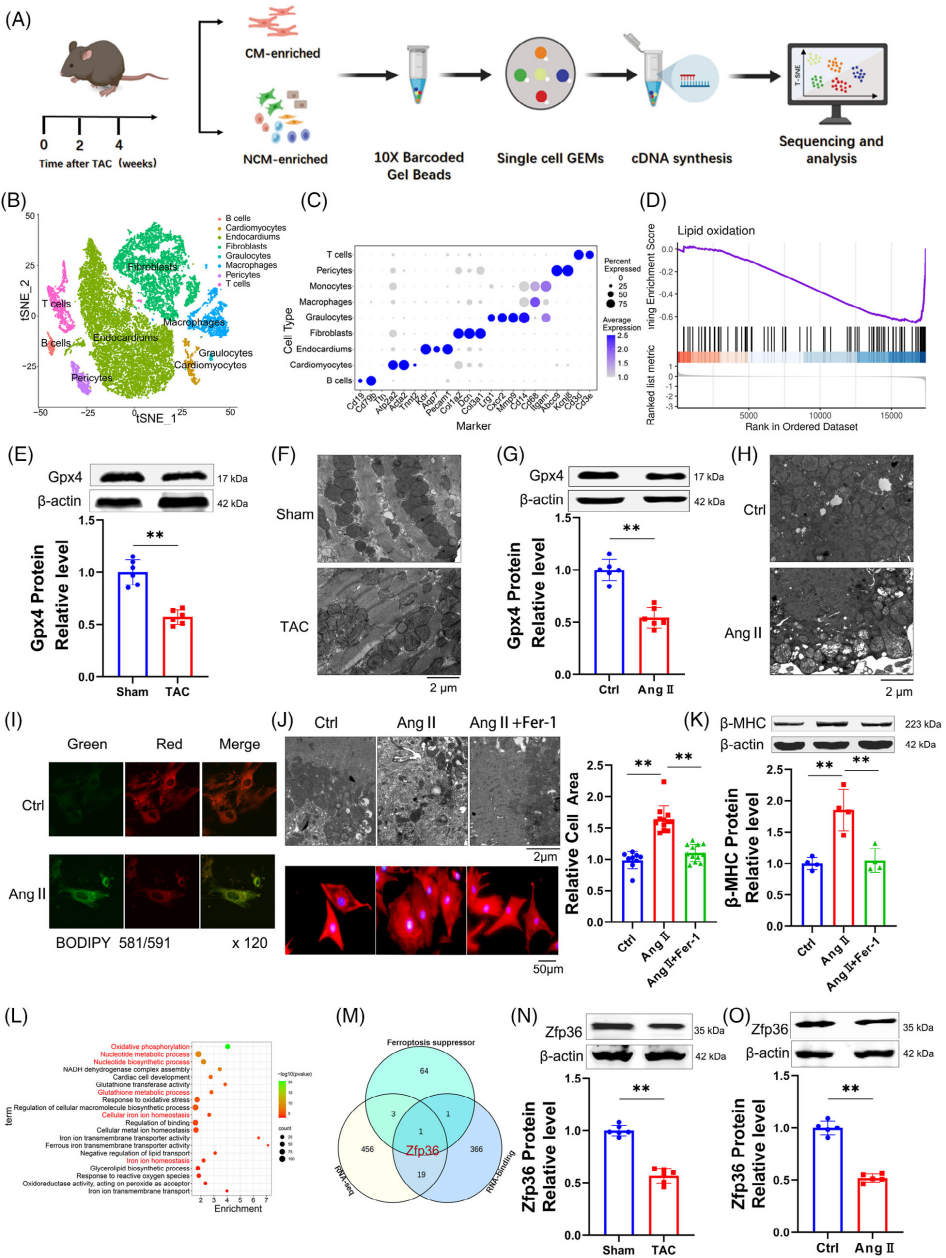
Ferroptosis‐associated zinc finger protein 36 (Zfp36) expression is reduced under cardiac hypertrophy. (A) Schematic of study design, and workflow of Nanogrid single‐cell selection and sequencing platform. (B) t‐Distributed stochastic neighbour embedding (t‐SNE) projection of cardiac tissue cells analysed by single‐cell RNA sequencing (RNA‐seq). Cells are coloured by distinct cell populations as indicated. (C) Top two to three distinct genes for each cell population, identified using an unsupervised approach. (D) Gene Set Enrichment Analysis (GSEA) showing that the genes related to lipid oxidation was upregulated in transverse aortic constriction (TAC) mice. (E and G) Western blot shown the protein expression level Gpx4 (*n* = 6). (F and H) Representative images of transmission electron microscope. (I) BODIPY581/591‐C11 probe staining for lipid peroxidation in cardiomyocytes after treating with Ang II. (J) Representative images of transmission electron microscope and cell area stained by α‐actinin (red) in cardiomyocytes. Nuclei were stained with 4',6‐diamidino‐2‐phenylindole (DAPI) (blue). Scale bars: 50 µm. Right row was the averaged data of cell area stained by α‐actinin (*n* = 9‒12). (K) Western blot results shown the protein expression level of beta‐myosin heavy chain (β‐MHC) (*n* = 4). (L) Gene Ontology (GO) analysis using significantly changed genes of RNA‐seq. (M) Venn diagram of ferroptosis suppressor, RNA‐binding protein and significantly changed genes shown in cardiac hypertrophy. (N‒O) Western blot results showed the expression level of Zfp36 (*n* = 5‒6). Statistical analysis was performed with Student's *t*‐test or one‐way analysis of variance (ANOVA). Results presented as mean ± standard deviation (SD). ^**^ *p* < .01.

Cardiac hypertrophy was induced by transverse aortic constriction (TAC), with a sham operation for the control group (Figure S1). To elucidate the molecular mechanisms underlying cardiac hypertrophy, single‐cell RNA sequencing (RNA‐seq) was conducted on cardiac tissues at 0, 2 and 4 weeks post‐TAC‐induced pressure overload (Figure 1A). Utilising clustering results from 25 328 single cells, the stochastic dimensionality reduction technique, t‐distributed stochastic neighbour embedding (t‐SNE), was employed to visualise the classification of single‐cell subpopulations (Figure 1B). Clustering analysis based on established marker genes identified a diverse array of cell types across all samples (Figure 1C). Our analysis concentrated on 679 single cardiomyocytes, from which transcriptomic data were obtained (Figure S2A,B). The transcriptomic profiles of cardiomyocytes exhibited significant alterations following the TAC procedure (Figure S2C). Gene Set Enrichment Analysis (GSEA) conducted on cardiomyocyte transcriptomes identified significant alterations in ferroptosis‐related signalling pathways, particularly those linked to reactive oxygen species (ROS) (Figure S2D) and lipid oxidation (Figure 1D). Notably, there was an elevated expression of Gpx4, a key suppressor of ferroptosis, within the cardiomyocytes (Figure S2E). Taken together, these findings suggest the activation of ferroptosis in the context of cardiac hypertrophy.

To investigate the activation of ferroptosis in cardiac hypertrophy, we analysed several ferroptosis‐associated markers, including malondialdehyde (MDA), prostaglandin‐endoperoxide synthase 2 (Ptgs2), Gpx4, and mitochondrial morphology. In TAC mice, there was a significant increase in MDA and Ptgs2 levels (Figure S2F,G), accompanied by a decrease in Gpx4 expression compared to sham‐operated mice (Figure 1E). Transmission electron microscopy revealed that the mitochondria in TAC mice displayed irregular arrangement, reduced size, thicker membranes and a darker appearance relative to those in sham mice (Figures 1F and S2H). Neonatal ventricular myocytes cultured with 1 µM Angiotensin II (Ang II) to induce hypertrophy (Figure S3A‒E) exhibited alterations in MDA (Figure S3F), Ptgs2 (Figure S3G), Gpx4 (Figure 1G) and mitochondrial characteristics (Figures 1H and S3H) that paralleled those observed in TAC mice. Furthermore, Ang II treatment resulted in increased lipid ROS and global ROS in cardiomyocytes (Figures 1I and S3I). These results suggest a potential role for ferroptosis cardiac hypertrophy.

To clarify the involvement of ferroptosis in cardiac hypertrophy, Ferrostatin‐1 (Fer‐1), a ferroptosis inhibitor, was utilised. The co‐incubation of Fer‐1 with Ang II significantly reduced cell surface area and mitigated mitochondrial alterations compared to treatment with Ang II alone (Figures 1J and S3J). Additionally, Fer‐1 significantly counteracted the increase in hypertrophic markers such as atrial natriuretic peptide (ANP), brain natriuretic peptide (BNP) and beta‐myosin heavy chain (β‐MHC) caused by Ang II (Figures 1K and S3K). These results suggest a contributory role of ferroptosis in cardiac hypertrophy. GSEA further identified a significant association between RNA binding and cardiac hypertrophy (Figure S3L). Given the limitations of current single‐cell sequencing technologies in detecting cell size, we utilised RNA‐seq to investigate differential genes in heart tissues from both sham and TAC groups. Gene Ontology (GO) analysis indicated that these genes were enriched in pathways related to ferroptosis and RNA biological regulation (Figure 1L). We conducted a Venn analysis utilizing the enrichment pathway gene set, the RBPs functional gene set (GO: 0003729), and a ferroptosis inhibitor gene set from the FerrDb database. The findings from our analysis indicate that the RBP Zfp36 may play a role in the regulation of myocardial hypertrophy (Figure 1M). Furthermore, the expression levels of Zfp36 protein were observed to be downregulated in hypertrophic conditions (Figure 1N,O), proposing that Zfp36 may be engaged in the ferroptosis process during cardiac hypertrophy.

### 2.2 Over‐expression of Zfp36 attenuates cardiac hypertrophy by inhibiting ferroptosis

**FIGURE 2:**
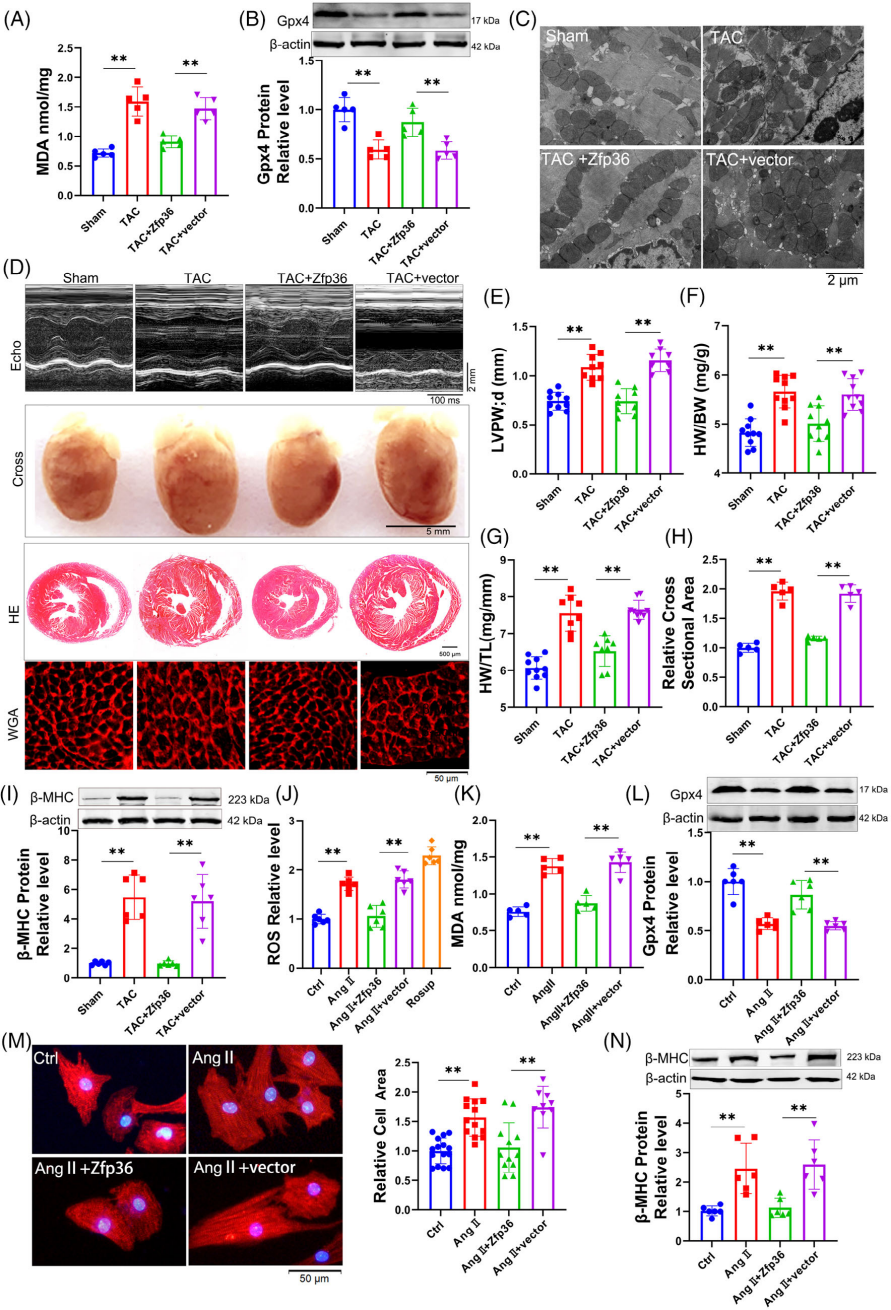
Zinc finger protein 36 (Zfp36)‐attenuated ferroptosis in cardiac hypertrophy. (A) Detection of malondialdehyde (MDA) for lipid peroxidation level in Sham and transverse aortic constriction (TAC) mice hearts (*n* = 5). (B) Western blot results shown the protein expression level of Gpx4 in mice hearts (*n* = 5). (C) Representative images of transmission electron microscope in mice hearts. (D) Representative images of echocardiography recording, heart cross‐morphology, heart sections stained with haematoxylin and eosin (HE) and wheat germ agglutinin (WGA) in mice. (E) Left ventricular posterior wall thickness dimensions during diastole in indicated mice (*n* = 6‒7). (F and G) Heart weight‐to‐body weight ratio and heart weight‐to‐tibia length ratio in mice (*n* = 8‒10). (H) Quantification of myocyte area from WGA staining heart sections (*n* = 5). (I and N) Western blot results shown the protein expression level of beta‐myosin heavy chain (β‐MHC) (*n* = 6). (J) DCFH‐DA probe determined the levels of reactive oxygen species (ROS) in cardiomyocytes (*n* = 6). (K) Detection of MDA for lipid peroxidation level (*n* = 5‒6). (L) Western blot analysed the expression levels of Gpx4 (*n* = 6). (M) Representative photographs of cardiomyocytes identified with α‐actinin antibody and the averaged data of cell area (*n* = 9‒15). Statistical analysis was performed with Student's *t*‐test or one‐way analysis of variance (ANOVA). Results presented as mean ± standard deviation (SD). ^**^ *p* < .01.

To evaluate the impact of Zfp36 on cardiac hypertrophy, Zfp36 was effectively overexpressed using an AAV9 vector driven by the cTnT promoter (Figure S4A). The overexpression of Zfp36‐attenuated TAC‐induced ferroptosis, as evidenced by reduced MDA levels (Figure 2A), decreased mRNA levels of Ptgs2 (Figure S4B), increased expression of Gpx4 (Figure 2B), alongside mitochondrial alterations (Figures 2C and S4C). Echocardiographic assessments demonstrated a thinner left ventricular (LV) wall in TAC mice treated with AAV9‐Zfp36 compared to those treated with the AAV9 vector alone (Figure 2D,E). Furthermore, reductions in heart size (Figure 2D), heart weight‐to‐body weight ratio (Figure 2F), heart weight‐to‐tibia length ratio (Figure 2G) and improved heart function (Figure S4D,E) were observed in hypertrophic hearts overexpressing Zfp36 compared to those with the vector. Histological examination using haematoxylin and eosin (HE) and wheat germ agglutinin (WGA) staining revealed smaller cross‐sectional areas of hearts and cardiomyocytes in mice overexpressing Zfp36 compared to vector‐treated mice following TAC (Figure 2D,H). Additionally, Zfp36 overexpression reversed the TAC‐induced upregulation of ANP, BNP and β‐MHC (Figures 2I and S4F).

To confirm the effects of Zfp36 on ferroptosis and hypertrophy in vitro, Zfp36 was overexpressed using a plasmid carrying Zfp36 (Figure S4G). Zfp36 has been shown to suppress Ang II‐induced ferroptosis responses, effectively reversing the upregulation of ROS and MDA (Figure 2J,K), as well as the downregulation of Gpx4 (Figure 2L). Furthermore, the overexpression of Zfp36 significantly mitigated the increase in cell size (Figure 2M) and attenuated the upregulation of hypertrophic markers, including ANP, BNP and β‐MHC, induced by Ang II (Figures 2N and S4H). Collectively, these findings suggest that Zfp36 overexpression alleviates cardiac hypertrophy through the inhibition of ferroptosis.

### 2.3 Zfp36 deficiency aggravates cardiac hypertrophy by promoting ferroptosis

**FIGURE 3:**
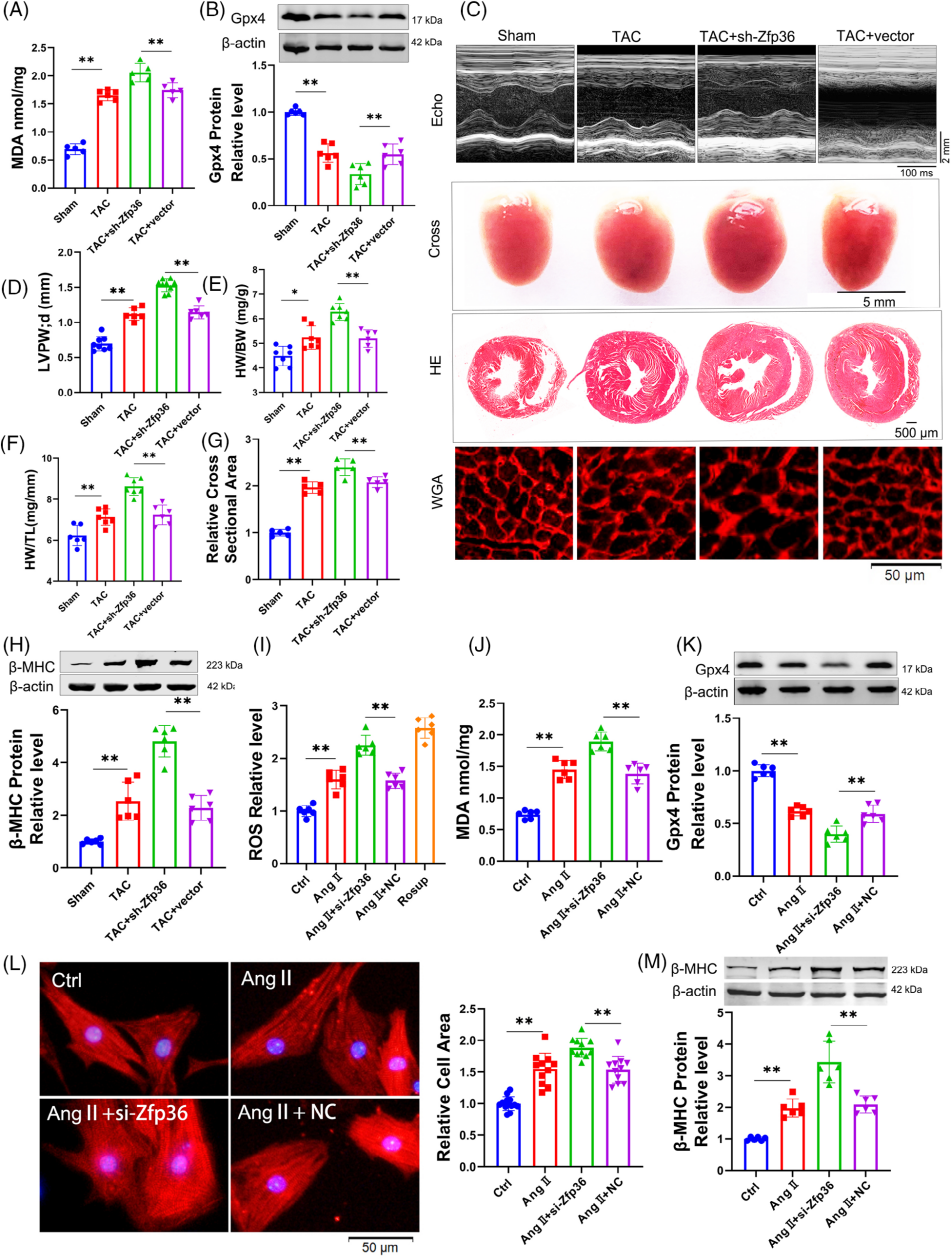
Knockdown zinc finger protein 36 (Zfp36) aggravate cardiac hypertrophy and ferroptosis. (A and J) Detection of malondialdehyde (MDA) for lipid peroxidation levels (*n* = 5‒6). (B and K) The expression level of Gpx4 protein (*n* = 6). (C) Representative images of echocardiography recording, heart cross‐morphology, heart sections stained with haematoxylin and eosin (HE) and wheat germ agglutinin (WGA) in mice. (D) Left ventricular wall thickness of mice (*n* = 5‒7). (E and F) Ratio of heart weight‐to‐body weight and heart weight‐to‐tibia length in mice (*n* = 6‒7). (G) Quantification of myocyte area from WGA staining heart sections (*n* = 5). (H and M) Western blot analysed the protein expression level of beta‐myosin heavy chain (β‐MHC) (*n* = 6). (I) DCFH‐DA probe determined the levels of reactive oxygen species (ROS) in cardiomyocytes (*n* = 6). (L) Representative photographs of cardiomyocytes identified with α‐actinin antibody and the averaged data of cell area (*n* = 11‒13). Statistical analysis was performed with one‐way analysis of variance (ANOVA). Results presented as mean ± standard deviation (SD). ^*^ *p* < .05; ^**^ *p* < .01.

To further elucidate the role of Zfp36 in cardiac hypertrophy and ferroptosis, AAV9 vectors carrying short hairpin RNA targeting Zfp36 (AAV9‐sh‐Zfp36), driven by the cardiac troponin T (cTnT) promoter, were employed to downregulate Zfp36 expression in cardiac tissues (Figure S4I). Notably, Zfp36‐deficient mice subjected to TAC exhibited elevated levels of MDA and Ptgs2 (Figures 3A and S4J), alongside a decrease in Gpx4 expression (Figure 3B) in comparison to TAC‐treated control mice. Additionally, echocardiographic analysis revealed a significant thickening of the LV wall in Zfp36‐deficient mice relative to control mice in response to TAC stimuli (Figure 3C,D). Moreover, Zfp36‐deficient mice demonstrated an increase in heart size (Figure 3C), as evidenced by elevated heart weight‐to‐body weight ratios (Figure 3E) and heart weight‐to‐tibia length ratios (Figure 3F) compared to control mice. Moreover, knocking down Zfp36 further aggravated cardiac decline (Figure S4K,L). Histological analyses using HE and WGA staining revealed larger cross‐sectional areas of hearts and cardiomyocytes in Zfp36‐deficient mice relative to controls (Figure 3C,G). These observations were corroborated by the upregulation of hypertrophic markers, including ANP, BNP and β‐MHC, in Zfp36‐deficient mice, indicating the presence of hypertrophic phenotypes (Figures 3H and S4M).

Subsequently, we investigated whether silencing Zfp36 could directly influence cardiomyocyte hypertrophy induced by Ang II in vitro. The expression of Zfp36 was silenced using small interfering RNA (siRNA) (Figure S4N). The results revealed that Ang II treatment resulted in elevated levels of ROS and MDA, accompanied by a reduction in the expression of Gpx4. These effects were further intensified by the application of si‐Zfp36 (Figure 3I‒K). Additionally, the siRNA‐mediated knockdown of Zfp36 exacerbated Ang II‐induced increases in hypertrophic markers, ANP, BNP, β‐MHC and cell size (Figures 3L,M and S4O). Collectively, these findings indicate that Zfp36 knockdown significantly exacerbates hypertrophy and ferroptosis in both in vivo and in vitro models.

To comprehensively elucidate the role of Zfp36 in the regulation of ferroptosis and hypertrophy, we employed advanced CRISPR‐Cas9 technology to construct an sgRNA plasmid targeting Zfp36 (sgZfp36) (Figure S5A). Successful knockout of Zfp36 was confirmed (Figure S5B,C), allowing us to assess whether the siRNA‐mediated promotion of ferroptosis and exacerbation of myocardial hypertrophy under Ang II conditions could be replicated. The findings demonstrated that sgZfp36 significantly increased ROS and MDA levels (Figure S5D,E) while reducing Gpx4 expression (Figure S5F) compared to the control group, thereby indicating that sgZfp36 enhances Ang II‐induced ferroptosis. Additionally, sgZfp36 was observed to upregulate the expression of hypertrophy‐related genes (Figure S5G,H) and enlarge cardiomyocyte area (Figure S5I). In summary, these results suggest that sgZfp36 facilitates ferroptosis and exacerbates cardiomyocyte hypertrophy, aligning with the effects observed with siRNA.

To investigate whether Zfp36 modulates cardiac hypertrophy through ferroptosis, we co‐incubated Fer‐1 with si‐Zfp36 to evaluate alterations in hypertrophic indices under conditions of cardiomyocyte hypertrophy. Our results demonstrated that Fer‐1 significantly mitigated the enlargement of cardiomyocyte area induced by si‐Zfp36 (Figure S6A) and reversed the increases in ANP, BNP and β‐MHC expression associated with si‐Zfp36 under Ang II treatment (Figure S6B,C). These observations indicate that Zfp36 influences the progression of cardiac hypertrophy through the regulation of ferroptosis.

### 2.4 Ythdc2 as a direct target of Zfp36 is upregulated in cardiac hypertrophy

**FIGURE 4:**
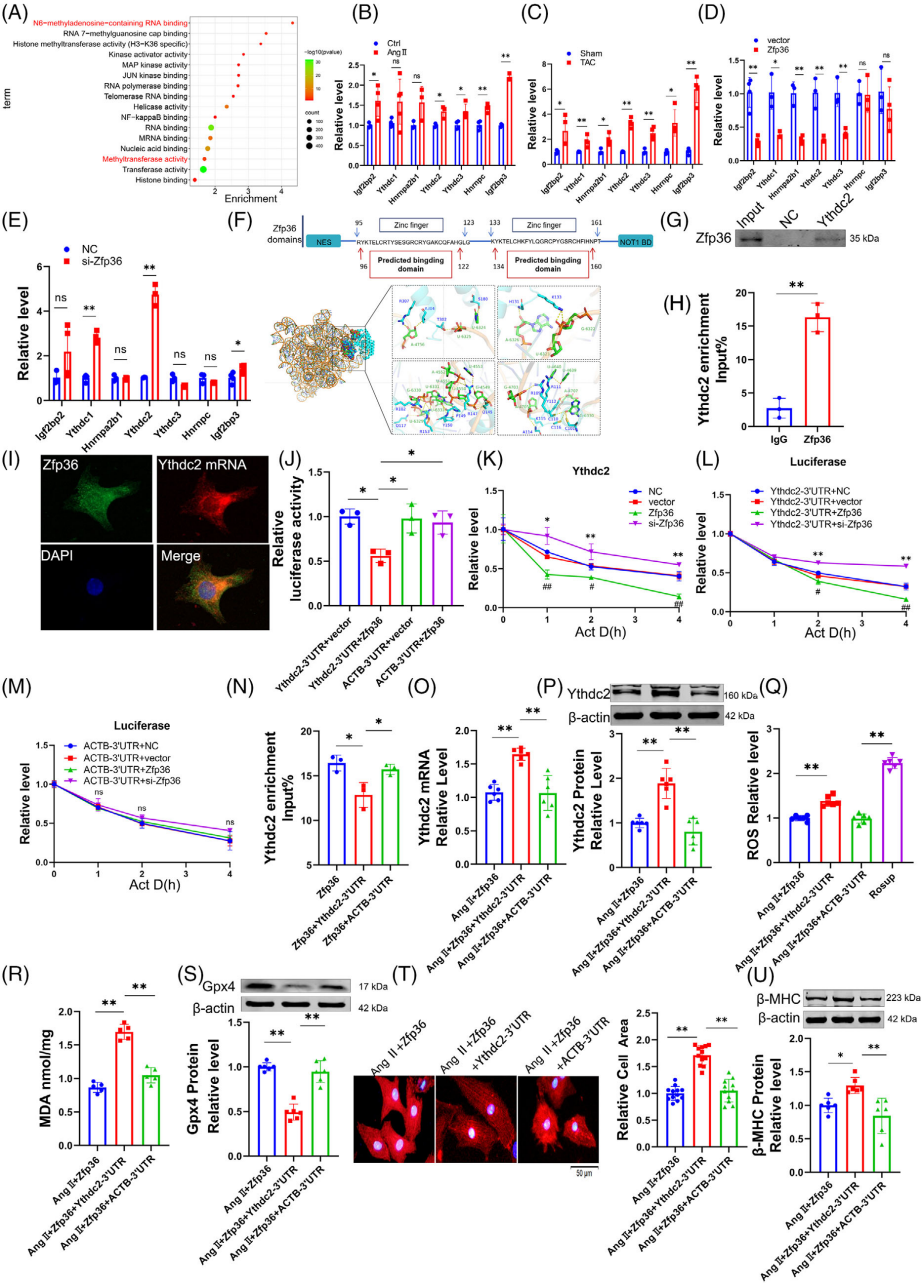
Ythdc2 as a direct target of zinc finger protein 36 (Zfp36) is upregulated in cardiac hypertrophy. (A) Gene Ontology (GO) analysis used the potential targets genes of Zfp36 and also exhibited high expression levels in RNA sequencing (RNA‐seq) data. (B‒E) qRT‐PCR analysed the mRNA expression levels of the potential targets of Zfp36 which were enriched in m^6^A regulating enzymes pathway (*n* = 3‒5). (F) Zfp36 protein structure diagram and the predicted binding region with Ythdc2 and docking results of Zfp36 protein with Ythdc2 3′ untranslated region (3′UTR) molecule, green chain (Ythdc2 3′UTR), the blue chain (Zfp36), the rod‐like structure represents the interacting amino acids and nucleic acids. (G) RNA‐pull down analysed the binding relation between Zfp36 and Ythdc2 mRNA. (H) RNA immunoprecipitation (RIP)‐PCR analysed the binding relation between Zfp36 and Ythdc2 mRNA (*n* = 3). (I) Co‐localisation of Zfp36 and Ythdc2 mRNA in cardiomyocyte. Zfp36 identified with Zfp36 antibody (green), Ythdc2 mRNA identified by its probes (red) and nuclei were stained with 4',6‐diamidino‐2‐phenylindole (DAPI) (blue). (J) Luciferase reporters of Ythdc2 3′UTR and ACTB 3′UTR in Hek293T cells transfected with increasing Zfp36 (*n* = 3). (K) qRT‐PCR analysed the mRNA expression levels of Ythdc2 treated with or not Act D in over‐expression or knockdown Zfp36 (*n* = 3‒6). (L and M) Over‐expression or knockdown Zfp36 CM was transfected with Ythdc2 3′UTR or ACTB 3′UTR luciferase plasmids, then subjected to analysis the expression level of luciferase mRNA at indicated time points treated with or not Act D (*n* = 4). (N) RIP‐PCR analysed the binding relation between Zfp36 and Ythdc2 mRNA (*n* = 3). (O and P) Real‐time PCR and western blotting analysed the expression levels of Ythdc2 (*n* = 6). (Q) DCFH‐DA probe staining for the reactive oxygen species (ROS) levels of cardiomyocytes (*n* = 6). (R) Detection of malondialdehyde (MDA) for lipid peroxidation level (*n* = 5). (S) Western blot results shown the protein expression level Gpx4 (*n* = 6). (T) The representative photographs of cardiomyocytes identified with α‐actinin and averaged data of cell area (*n* = 11‒12). (U) The protein expression level of beta‐myosin heavy chain (β‐MHC) (*n* = 6). Statistical analysis was performed with Student's *t*‐test or one‐way analysis of variance (ANOVA). Results presented as mean ± standard deviation (SD). ^*^ *p* < .05; ^**^ *p* < .01.

This section examines the targets of Zfp36 during cardiac hypertrophy. To identify potential downstream mRNA targets of Zfp36, the online tool ENCORI (https://starbase.sysu.edu.cn/index.php) was employed. Following this, a GO analysis was conducted on genes identified as potential Zfp36 targets by ENCORI, which also exhibited high expression levels in RNA‐seq data. The analysis revealed that some of these potential targets were enriched in pathways involving m^6^A‐regulating enzymes (Figure 4A). Additionally, we observed a significant upregulation of Ythdc2 expression in hypertrophic conditions, both in vitro and in vivo (Figures 4B,C and S7A,B). The expression levels of Ythdc2 increased with the downregulation of Zfp36 and decreased with its upregulation (Figures 4D,E and S7C,D). This inverse correlation between Ythdc2 and Zfp36 suggests a potential targeting relationship.

Previous studies have demonstrated that mutations within the TZF domain completely abolish RNA binding activity,^14^ highlighting its essential role in the biological function of Zfp36. Consistently, computational predictions using catRAPID (http://service.tartaglialab.com/page/catrapid_group) indicated binding of Zfp36 to Ythdc2 mRNA via the TZF domain (Figure 4F). Molecular docking analyses provided additional evidence supporting the interaction between Zfp36 and Ythdc2 mRNA (Figure 4F). Subsequent RNA immunoprecipitation (RIP) and pull‐down assays confirmed the co‐immunoprecipitation of Ythdc2 mRNA with the Zfp36 protein (Figure 4G,H). Immunofluorescence combined with fluorescence in situ hybridisation (IF‐FISH) staining further demonstrated the co‐localisation of Ythdc2 mRNA and Zfp36 protein within cardiomyocytes (Figure 4I). Consistently, Dual Luciferase Reporter Assays revealed that Zfp36 significantly decreased luciferase activity when linked to the Ythdc2 3′ untranslated region (3′UTR), but not when linked to the ACTB 3′UTR (Figure 4J), with the latter lacking Zfp36 binding sites.^20^ The half‐life of Ythdc2 mRNA was extended in cells with silenced Zfp36 expression and reduced in cells overexpressing Zfp36, highlighting Zfp36's role in the post‐transcriptional degradation of Ythdc2 mRNA (Figure 4K). Furthermore, Zfp36 specifically modulated the half‐life of luciferase mRNA associated with the Ythdc2 3′UTR, but not with the ACTB 3′UTR (Figure 4L,M), suggesting that the downregulation of Ythdc2 by Zfp36 occurs through its interaction with the Ythdc2 3′UTR.

To further elucidate the mechanism by which Zfp36 inhibits ferroptosis in cardiomyocyte hypertrophy through its interaction with Ythdc2, we conducted overexpression of Zfp36 and transfection of the Ythdc2 3′UTR under Ang II conditions. The results demonstrated that co‐transfection of Zfp36 and the Ythdc2 3′UTR led to a reduction in the binding affinity between Zfp36 and Ythdc2 mRNA (Figure 4N), which was accompanied by increased levels of Ythdc2 mRNA and protein expression (Figure 4O,P). Subsequently, we observed that the Ythdc2 3′UTR could counteract the Zfp36‐mediated reductions in ROS and MDA levels (Figure 4Q,R), enhance Gpx4 expression (Figure 4S), decrease cardiomyocyte area (Figure 4T) and reduce the expression of ANP, BNP and β‐MHC induced by Ang II (Figures 4U and S7E). These findings provide evidences that Zfp36 exerts regulatory effects on ferroptosis in cardiomyocytes by binding to Ythdc2 mRNA and facilitating its degradation.

### 2.5 Knockdown of Ythdc2 improves ferroptosis and mitigates cardiac hypertrophy

**FIGURE 5:**
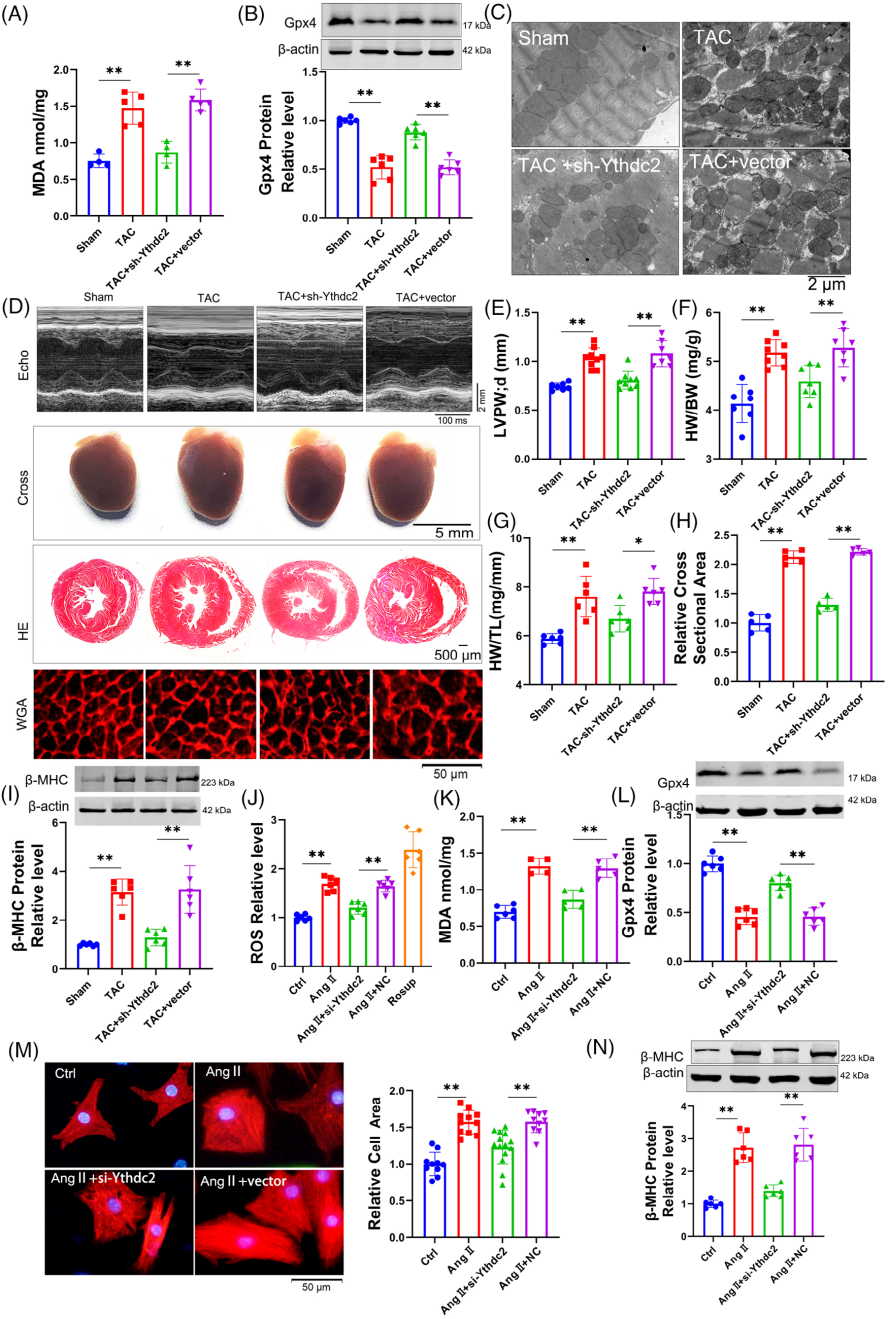
Knockdown Ythdc2‐attenuated ferroptosis in hypertrophy induced by transverse aortic constriction (TAC). (A and K) Detection of malondialdehyde (MDA) for lipid peroxidation level (*n* = 4‒5). (B and L) Western blot results show the protein expression level of Gpx4 (*n* = 6). (C) Representative images of transmission electron microscope in mice. (D) Representative images of echocardiography recording, heart cross‐morphology, heart sections stained with haematoxylin and eosin (HE) and wheat germ agglutinin (WGA) in mice. (E) Left ventricular posterior wall thickness dimensions during diastole in indicated mice (*n* = 7‒9). (F and G) Heart weight‐to‐body weight ratio and heart weight‐to‐tibia length ratio (*n* = 6‒8). (H) Quantification of myocyte area from WGA staining heart sections (*n* = 5). (I and N) Western blot results shown the protein expression level of beta‐myosin heavy chain (β‐MHC) (*n* = 6). (J) DCFH‐DA probe staining for the reactive oxygen species (ROS) levels of cardiomyocytes (*n* = 6). (M) Representative photographs and averaged data of cell area stained by α‐actinin antibody the averaged data of cell area (*n* = 10‒14). Statistical analysis was performed with one‐way analysis of variance (ANOVA). Results presented as mean ± standard deviation (SD). ^*^ *p* < .05; ^**^ *p* < .01.

To elucidate the role of Ythdc2 in ferroptosis and cardiac hypertrophy, we conducted a series of loss‐of‐function experiments. Mice were administered AAV9 viral particles containing sh‐Ythdc2 driven by the cTnT promoter via tail vein injection. The efficacy of sh‐Ythdc2 was confirmed through western blot analysis (Figure S8A). Depletion of Ythdc2 led to a reduction in MDA (Figure 5A) and Ptgs2 (Figure S8B) levels, an increase in Gpx4 levels (Figure 5B), and improvements in mitochondrial morphology compared to the vector control in TAC mice (Figures 5C and S8C). Furthermore, Ythdc2 knockdown attenuated the enlargement of heart size and weight, decreased LV wall thinning and improved heart function in TAC mice (Figures 5D‒G and S8D,E). Inhibition of Ythdc2 also partially impeded the enlargement of cardiomyocyte size (Figure 5H) and the expression of genes associated with cardiac hypertrophy (Figures 5I and S8F).

To further investigate the effects of Ythdc2 deficiency on cardiac hypertrophy, additional in vitro experiments were performed. Specifically, siRNA was employed to suppress Ythdc2 expression (Figure S8G), and its impact on hypertrophy and ferroptosis was assessed. The findings indicated that Ythdc2 suppression led to a significant reduction in the expression of ROS (Figure 5J), MDA (Figure 5K), as well as an increase of GPX4 expression (Figure 5L). Concurrently decreased in cell size in hypertrophic cardiomyocytes (Figure 5M) and hypertrophy‐related genes (Figures 5N and S8H). These results collectively demonstrate that Ythdc2 deficiency inhibits both ferroptosis and cardiac hypertrophy.

### 2.6 Ythdc2 exacerbates ferroptosis and cardiac hypertrophy

**FIGURE 6:**
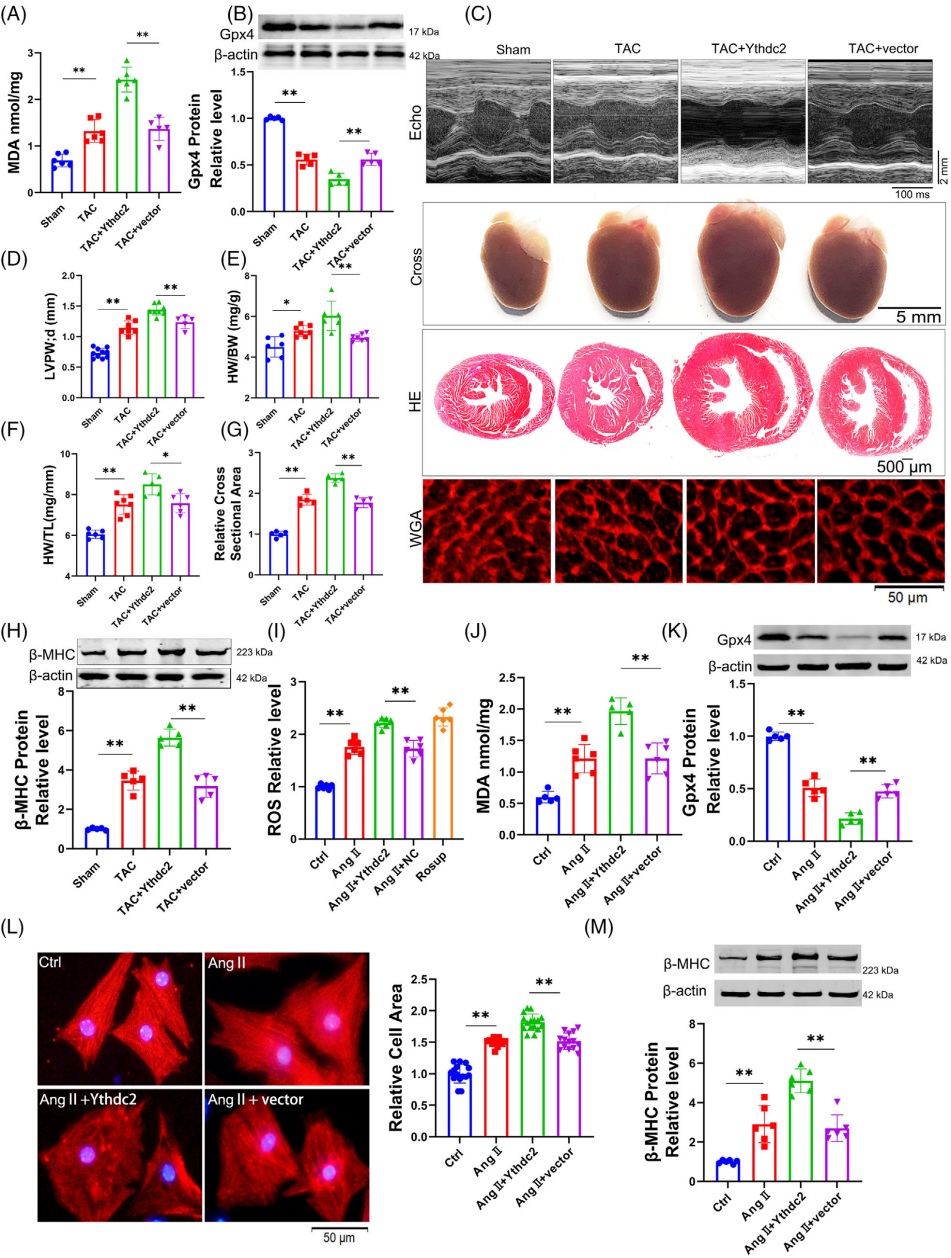
Ythdc2 exacerbates ferroptosis and cardiac hypertrophy. (A and J) Detection of malondialdehyde (MDA) for lipid peroxidation level (*n* = 5‒6). (B and K) Western blot results shown the protein expression level Gpx4 (*n* = 5). (C) Representative images of echocardiography recording, heart cross‐morphology, heart sections stained with haematoxylin and eosin (HE) and wheat germ agglutinin (WGA) in mice. (D) Left ventricular posterior wall thickness dimensions during diastole in indicated mice (*n* = 6‒7). (E and F) Heart weight‐to‐body weight ratio and heart weight‐to‐tibia length ratio (*n* = 5‒7). (G) Quantification of myocyte area from WGA staining heart sections (*n* = 5). (H and M) Western blot results show the protein expression level of beta‐myosin heavy chain (β‐MHC) (*n* = 5). (I) DCFH‐DA probe staining for the reactive oxygen species (ROS) levels of cardiomyocytes (*n* = 6). (L) The representative photographs and averaged data of cell area stained by α‐actinin antibody (red), nuclei were stained with 4',6‐diamidino‐2‐phenylindole (DAPI) (blue) (*n* = 13‒16). Statistical analysis was performed with one‐way analysis of variance (ANOVA). Results presented as mean ± standard deviation (SD). ^*^ *p* < .05; ^**^ *p* < .01.

In contrast, we undertook gain‐of‐function research wherein mice were administered AAV9 viral particles harboring Ythdc2. Western blot analysis confirmed the efficacy of Ythdc2 overexpression (Figure S8I). Ythdc2 overexpression exacerbated the elevation of MDA and Ptgs2 levels (Figures 6A and S8J), while concurrently reducing Gpx4 expression (Figure 6B) in comparison to vector‐treated TAC mice. Notably, functional and postmortem analyses demonstrated that Ythdc2 overexpression significantly increased cardiac dimensions, HW/BW, HW/TL (Figure 6C‒F) and reduced heart function (Figure S8K,L). Cross‐sectional analysis revealed enlarged cardiac and cardiomyocyte areas in Ythdc2‐overexpressing mice following TAC (Figure 6C,G). Correspondingly, the expression levels of cardiac hypertrophy markers were upregulated in these TAC mice (Figures 6H and S8M). Overexpression of Ythdc2 (Figure S8N) via a Ythdc2‐carrying plasmid in vitro similarly intensified increases in ROS (Figure 6I), MDA (Figure 6J) and decreased Gpx4 expression (Figure 6K). The cell area and hypertrophic markers increased further (Figures 6L,M and S8O). Collectively, these results provide compelling evidence that Ythdc2 plays a pivotal role in regulating both ferroptosis and cardiac hypertrophy.

### 2.7 Regulation of Ythdc2 expression by Zfp36 mediates ferroptosis and cardiac hypertrophy phenotypes

**FIGURE 7:**
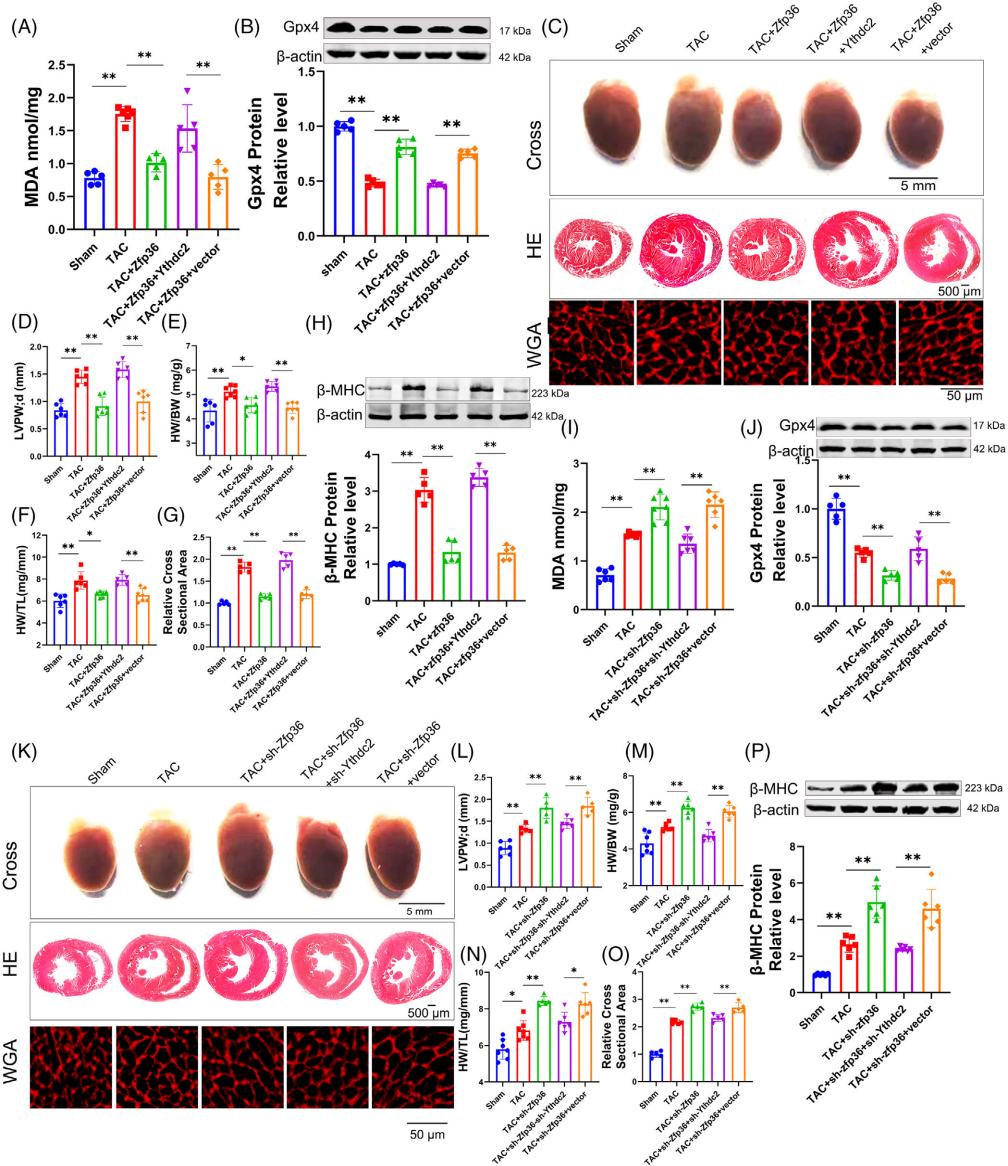
Regulated of Ythdc2 expression by zinc finger protein 36 (Zfp36) mediates the ferroptosis and cardiac hypertrophy phenotypes. (A and I) Detection of malondialdehyde (MDA) for lipid peroxidation level in mice hearts (*n* = 5‒7). (B and J) Western blot results shown the protein expression level Gpx4 (*n* = 5‒6). (C and K) Representative images of heart cross‐morphology, heart sections stained with haematoxylin and eosin (HE) and wheat germ agglutinin (WGA) in mice. (D and L) Left ventricular (LV) posterior wall thickness dimensions in indicated mice (*n* = 5‒7). (E and M) Heart weight‐to‐body weight ratio (*n* = 6‒7). (F and N) Heart weight‐to‐tibia length ratio (*n* = 5‒7). (G and O) Quantification of myocyte area from WGA stained (*n* = 5). (H and P) Protein expression level of beta‐myosin heavy chain (β‐MHC) (*n* = 5‒6). Statistical analysis was performed with one‐way analysis of variance (ANOVA). Results presented as mean ± standard deviation (SD). ^*^ *p* < .05; ^**^ *p* < .01.

The initial findings indicated that Zfp36 is involved in mitigating ferroptosis during cardiac hypertrophy by targeting Ythdc2. To further elucidate this relationship, subsequent experiments were conducted to evaluate the impact of Zfp36 on the regulation of Ythdc2 in the context of cardiac hypertrophic growth and ferroptosis. As illustrated in Figures 7 and S9, the overexpression of Ythdc2 impaired the ability of Zfp36 to counteract TAC/Ang II‐induced ferroptosis and hypertrophy. This was demonstrated by increased levels of MDA and Ptgs2 (Figures 7A and S9A), and decreased cardiac Gpx4 levels (Figure 7B), enlarged heart size (Figure 7C‒G), upregulation of hypertrophy‐associated genes (Figure 7H) in TAC mice. Consistent results were observed in vitro: co‐overexpression of Ythdc2 and Zfp36 under Ang II conditions resulted in elevate ROS and MDA levels (Figure S9B,C), reduced Gpx4 expression (Figure S9D), increased cell size (Figure S9E) and a higher hypertrophy index compared to Zfp36 overexpression alone (Figure S9F,G).

In contrast, the knockdown of Ythdc2 counteracted the upregulation of ANP, BNP and β‐MHC induced by sh‐Zfp36 or si‐Zfp36 during TAC/Ang II‐induced ferroptosis and hypertrophy. This reversal was associated with reductions in levels of ptgs2 (Figure S9H), ROS (Figure S9I) and MDA (Figures 7I and S9J), and enhancements in Gpx4 levels (Figures 7J and S9K). The heart size and cell size were downregulated (Figures 7K‒O and S9N). The expression of hypertrophy‐related genes (Figures 7P and S9L,M). Together, these findings indicate that Zfp36 modulates cardiac hypertrophy and ferroptosis through its interaction with Ythdc2.

### 2.8 Zfp36 through Ythdc2/SLC7A11/GSH‐dependent ferroptosis pathway improve cardiac hypertrophy

**FIGURE 8:**
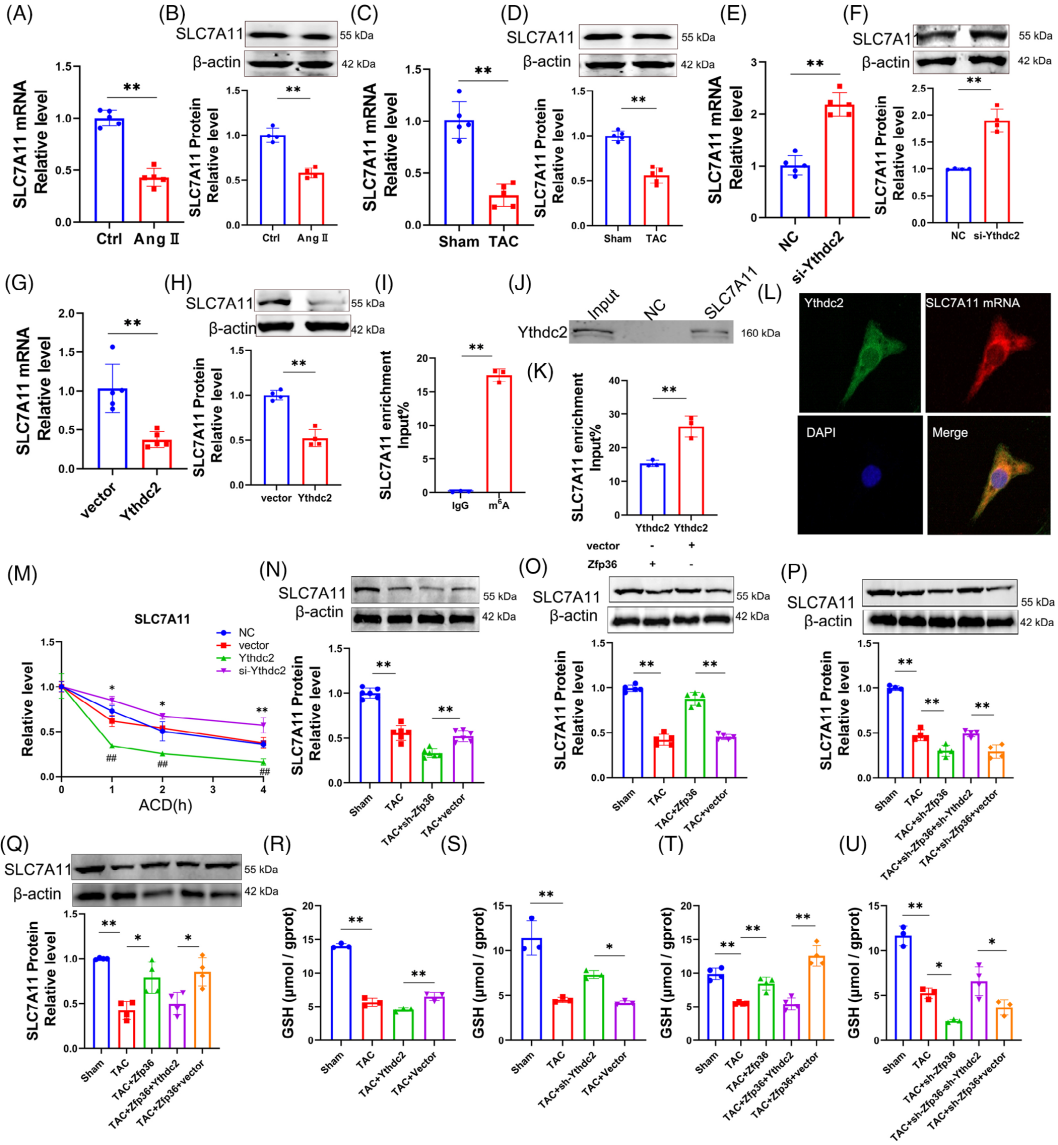
Zinc finger protein 36 (Zfp36) through the Ythdc2/SLC7A11/glutathione (GSH)‐dependent ferroptosis pathway improve cardiac hypertrophy. (A‒H) The mRNA and protein expression levels of SLC7A11 (*n* = 4‒5). (I) RNA immunoprecipitation (RIP)‐PCR analysed the m^6^A modification of SLC7A11 mRNA (*n* = 3). (J) RNA‐pull down verified Ythdc2 bond to SLC7A11 mRNA (*n* = 3). (K) RIP analysed the binding relation between Ythdc2 and SLC7A11 mRNA (*n* = 3). (L) Co‐localisation of Ythdc2 and SLC7A11 mRNA in cardiomyocyte. (M) Real‐time PCR analysed the mRNA expression levels of SLC7A11 treated with or not Act D in over‐expression or knockdown Ythdc2 (*n* = 3‒4). (N‒Q) Protein expression levels of SLC7A11 in mice hearts (*n* = 4‒6). (R and S) GSH was negatively regulated by Ythdc2 in cardiac hypertrophy (*n* = 3). (T‒U) Zfp36 regulates GSH level by Ythdc2 (*n* = 3‒4). Statistical analysis was performed with Student's *t*‐test or one‐way analysis of variance (ANOVA). Results presented as mean ± standard deviation (SD). ^*^ *p* < .05; ^**^ *p* < .01.

Previous research has demonstrated that SLC7A11 mRNA undergoes m6A modification, with the YTH domain of Ythdc2 facilitating m6A‐dependent degradation of SLC7A11 mRNA.^21^ SLC7A11 serves as a cystine/glutamate antiporter, playing a crucial role in mediating cysteine transport for Gpx4 and acting as a key inhibitor of ferroptosis.^21^ This study examines the regulatory influence of Ythdc2 on SLC7A11 within the context of cardiac hypertrophy. Both SLC7A11 mRNA and protein levels were observed to decrease during hypertrophy, with Ythdc2 exerting a negative regulatory effect (Figure 8A‒H). A putative m^6^A motif was identified within the SLC7A11 mRNA sequence (Figure 8I), and an interaction between SLC7A11 mRNA and Ythdc2 protein was confirmed (Figure 8J‒L). Furthermore, Ythdc2 overexpression was found to accelerate SLC7A11 mRNA degradation, whereas Ythdc2 inhibition mitigated this degradation (Figure 8M). These findings underscore the critical role of Ythdc2 in promoting SLC7A11 mRNA degradation in cardiomyocytes.

Moreover, Zfp36 has been identified as a key regulator of SLC7A11 protein expression, with Ythdc2 having the capacity to counteract the influence of Zfp36 on SLC7A11 expression (Figure 8N‒Q). SLC7A11 plays a critical role in GSH biosynthesis and antioxidant defense.^22^ Our GSH assays revealed diminished GSH levels in cardiac hypertrophy, which were further exacerbated by Ythdc2 overexpression and mitigated by Ythdc2 knockdown (Figure 8R,S). Furthermore, Zfp36 overexpression led to an increase in GSH levels; however, this effect was negated by concurrent Ythdc2 overexpression in the context of cardiac hypertrophy (Figure 8T). The knockdown of Zfp36 resulted in a further reduction of GSH content, a condition that was reversed by Ythdc2 knockdown in TAC mice (Figure 8U). Collectively, these findings highlight the role of Zfp36 in modulating cardiac hypertrophy via the Ythdc2/SLC7A11/GSH‐dependent ferroptosis pathway.

## 3 DISCUSSION

The data reveals the critical role of Zfp36, a protein responsive to stress conditions, in modulating the outcomes of cardiac hypertrophy. Our results demonstrate that Zfp36 mitigates cardiac ferroptosis and attenuates hypertrophy by facilitating the degradation of Ythdc2 mRNA, Ythdc2 in turn restores SLC7A11 expression. This study provides compelling evidence, for the first time, that Zfp36 is integral in suppressing ferroptosis and reducing cardiac hypertrophy through the Ythdc2/SLC7A11/GSH axis in cardiomyocytes (Graphical Abstract). These findings propose a novel therapeutic approach for managing cardiac hypertrophy by targeting ferroptosis.

The onset of cardiac hypertrophy initiates downstream signalling cascades, leading to an increased accumulation of intracellular ROS and intermediates. This accumulation subsequently results in cell death, fibrosis, calcium imbalance, mitochondrial dysfunction, insufficient angiogenesis and other biological processes, thereby exacerbating cardiac hypertrophy.^23^, ^24^ Previous research has implicated ferroptosis in the development of cardiac hypertrophy.^25^ This hypothesis is further corroborated by our single‐cell RNA‐seq data. Molecular analyses conducted on hypertrophic mouse models and cell cultures demonstrated decreased levels of Gpx4, alongside increased levels of Ptgs2 and MDA. Notably, the inhibition of ferroptosis using Fer‐1 significantly attenuated the hypertrophic response induced by Ang II in cardiomyocytes. These findings collectively highlight the potential therapeutic advantage of targeting ferroptosis to mitigate cardiac hypertrophy.

Indeed, ferroptosis has been identified as a critical trigger for cardiac hypertrophy.^11^ The hallmark characteristics of ferroptosis include disruptions in iron metabolism, accumulation of ROS, and lipid peroxidation, all of which are intricately linked to the onset and progression of myocardial hypertrophy.^12^, ^26^ The increase in cytoplasmic iron associated with ferroptosis can interfere with Ca^2+^ transport in cardiomyocytes, impairing excitation‒contraction coupling and resulting in compensatory hypertrophy and heart failure.^27^ Furthermore, lipid peroxidation leads to the disruption of cell membranes, including those of mitochondria, causing mitochondrial dysfunction that regulates cardiac hypertrophy.^28^, ^29^ In addition, ROS are implicated in promoting myocardial hypertrophy through the activation of various signalling pathways, including the PI3K/Akt and MAPK pathways, which influence gene expression and the proliferation of cardiomyocytes.^30^ ROS can impact the contractile function of cardiomyocytes by modulating the activity of Ca^2+^ channels and altering intracellular calcium ion concentrations.^31^, ^32^ These findings substantiate our experimental conclusion that targeting ferroptosis, novel programmed cell death, can ameliorate myocardial hypertrophy.

Recent research has demonstrated that various forms of programmed cell death, including ferroptosis, apoptosis and necrosis, play a role in the regulation of myocardial hypertrophy.^12^ Importantly, these forms of cell death do not occur in isolation; rather, they interact with one another and contribute to pathophysiological processes.^33^ Studies have indicated that targeted inhibition of AKT can reduce apoptosis and ferroptosis.^34^ Furthermore, another study has confirmed that ferroptosis can trigger pyroptotic signalling in heart failure induced by TAC.^10^ Given that the disruption of homeostasis may activate multiple pathways leading to cell death,^35^ a comprehensive investigation into the modes and mechanisms of cell death could facilitate the development of novel therapeutic strategies.

The primary discovery of our study is the regulatory role of Zfp36 in ferroptosis within the context of cardiac hypertrophy. Zfp36 destabilises ARE‐containing mRNAs, which are essential for post‐transcriptional regulation in both development and disease.^13^ Recognised for its capacity to modulate cytokines and chemokines, Zfp36 function as a broad anti‐inflammatory agent by reducing mRNA stability, thereby affecting immune responses.^14^ Initial investigations associated with hematopoietic alterations and the suppression of inflammation changes in Zfp36 expression in early‐stage myocardial hypertrophy models.^36^ Moreover, Zfp36 has been associated with the inhibition of ferroptosis in various diseases,^37^ although its precise role in ferroptosis‐mediated cardiac hypertrophy requires further exploration. In this investigation, multiple lines of evidence were generated to clarify the function of Zfp36 in ferroptosis and cardiac hypertrophy. Initially, bioinformatics analyses of single‐cell and RNA‐seq revealed that Zfp36 may inhibit ferroptosis during the cardiac hypertrophy process. Additionally, a significant downregulation of Zfp36 was observed in hypertrophied myocardium in both in vivo and in vitro models. Moreover, we demonstrate that the overexpression of Zfp36 significantly mitigates cardiac hypertrophy induced by Ang II and TAC by alleviating ferroptosis, whereas the knockdown exacerbates these pathological conditions. The underlying mechanisms governing the alteration of Zfp36 expression in cardiomyocytes during myocardial hypertrophy have yet to be elucidated, presenting an ongoing challenge for clinical application. Recent research has demonstrated that MAPK signalling influences the de novo transcription of Zfp36 mRNA in the context of VEGF‐stimulated developmental retinal angiogenesis.^38^ Furthermore, single‐cell sequencing analysis of dilated cardiomyopathy has revealed that Zfp36 is modulated by the transcription factor AP‐1.^17^

Mechanistically, our findings reveal that Zfp36 interacts with Ythdc2 mRNA, promoting its degradation. Ythdc2, an m^6^A reader, participates in various physiological and pathological processes via m^6^A‐dependent pathways.^39^ Ythdc2 has been identified as a destabilising agent for SLC7A11 mRNA, a critical suppressor of ferroptosis, through an m^6^A‐dependent mechanism, thereby facilitating its degradation. This process subsequently hinders cysteine uptake and disrupts antioxidative pathways, resulting in ferroptosis.^21^ SLC7A11 functions as the transporter subunit in the System X_c_^−^‐mediated uptake of cystine, an oxidised form of cysteine that is later reduced to cysteine in the cytosol. Cysteine acts as a rate‐limiting precursor alongside glycine and glutamate to synthesise GSH, which serves as a cofactor for Gpx4.^22^ Gpx4 uniquely possesses the ability to change PL hydroperoxides into PL alcohols. It can inhibit ferroptosis and oxidative damage caused by ROS accumulation and lipid peroxidation. It has been reported that the RBP RBMS1 negatively influences the translation of SLC7A11, thereby promoting ferroptosis. This regulatory mechanism is pertinent in the context of cardiovascular diseases, as demonstrated by SLC7A11's role in preventing Ang II‐induced cardiac hypertrophy through ferroptosis inhibition.^40^

These findings indicate that targeting the Zfp36/Ythdc2/SLC7A11 axis could protect against myocardial hypertrophy. Potential strategies include developing Zfp36 peptide drugs or using antisense RNA to prevent Zfp36 mRNA degradation. RNA‐based therapies, such as vaccines and antisense oligonucleotides, have been effective in other diseases. Additionally, Ythdc2 inhibitors or Zfp36 agonists could be explored. Key challenges for clinical application include targeting issues, drug delivery, and the need for targeted vectors to enhance drug entry into target organs.

## 4 CONCLUSIONS

Ferroptosis plays a significant role in the regulation of myocardial hypertrophy. Our findings reveal that Zfp36 ameliorates cardiac hypertrophy by inhibiting ferroptosis. Specifically, Zfp36 binds to Ythdc2 mRNA and facilitates its degradation. Ythdc2 interacts with SLC7A11 mRNA, facilitating its degradation and resulting in decreased GSH levels, thereby modulating ferroptosis in cardiomyocytes and influencing cardiac hypertrophy. This study may offer a novel theoretical framework and innovative strategies for a comprehensive understanding and clinical management of cardiac hypertrophy.

## 5 MATERIAL AND METHODS

### 5.1 Animals and treatment

All animal handling protocols were approved by the Animal Care and Use Committee of Harbin Medical University (IRB5017622). All animal procedures conform to the guidelines from Directive 2010/63/EU of the European Parliament on the protection of animals used for scientific purposes or the NIH Guide for the Care and Use of Laboratory Animals.

Healthy adult C57BL/6 male mice (6‒8 weeks, weighing 20–25 g), purchasing from Liaoning Changsheng Biotechnology Co. Ltd. were used. The mice were given injections of either adeno‐associated virus AAV9‐sh‐Zfp36, AAV9‐Zfp36, AAV9‐sh‐Ythdc2, AAV9‐Ythdc2 or AAV9‐vector (Hanbio). After 3 weeks, pressure‐overload model was performed for 4 weeks to establish a mouse model of cardiac hypertrophy.

The pressure‐overload cardiac hypertrophy model was developed using TAC in mice. Mice were anaesthetised with 2% tribromoethanol via intraperitoneal injection, confirmed by the absence of a tail pinch reflex. They were placed supine, intubated and connected to a ventilator (Ugo Basile SRL3). The pressure‐overload cardiac hypertrophy model was developed using TAC in mice. Mice were anaesthetised with 2% tribromoethanol via intraperitoneal injection, confirmed by the absence of a tail pinch reflex. They were placed supine, intubated and connected to a ventilator.

### 5.2 Echocardiography

At the end of TAC (2 or 4 weeks), mice were lightly anaesthetised with 2% tribromoethanol were placed on a platform. Cardiac anatomical and functional parameters were evaluated by parasternal short‐axis images acquired in M‐mode using the Visual Sonic Ultrasound system (vevo2100) and to determine wall thickness, and end‐diastolic and end‐systolic dimensions. LV wall thickness was used as an index of cardiac hypertrophy.

### 5.3 Tissue dissociation and preparation of single‐cell suspensions

We obtained three myocardial tissue samples from each of the three distinct experimental groups: the sham operation, TAC 2 weeks and TAC 4 weeks group. Three heart muscle tissues were taken from each group as a whole for single‐cell sequencing. Mice were euthanised by cervical dislocation and heart tissues were obtained from sham and TAC 2 weeks and TAC 4 weeks mice. Place a sterile RNase‐free culture dish containing an appropriate amount of calcium‐free and magnesium‐free phosphate buffered saline (PBS) on ice. Tissue dissociated and single‐cell suspension prepared by LC‐Bio Technology Co. Ltd.

### 5.4 Chromium 10× genomics library and sequencing

Single‐cell suspensions were loaded to 10× chromium according to the manufacturer's instructions of 10× Genomics Chromium Single‐Cell 3′kit (V3). The following cDNA amplification and library construction steps were performed according to the standard protocol. Libraries were sequenced on an Illumina NovaSeq 6000 sequencing system (paired‐end multiplexing run, 150 bp) by LC‐Bio Technology Co. Ltd. at a minimum depth of 20 000 reads per cell.

### 5.5 Data processing and data visualisation

The Illumina bcl2fastq software (v2.20) was used to demultiplex sequencing data and convert it to FASTQ format. The Cell Ranger pipeline (v6.1.1) processed barcodes, counted single‐cell 3′ genes, and aligned single‐cell RNA‐seq data to the GRCh38 reference genome. The output was analysed using Seurat (v3.1.1) for dimensionality reduction and clustering in R (v3.5.2). Single‐cell data analysis was conducted with OmicStudio tools by LC‐Bio Technology at https://www.omicstudio.cn/cell.

### 5.6 Culture of neonatal ventricular cardiomyocytes

Neonatal ventricular cardiomyocytes were isolated from the hearts of neonatal P1 or P3 Kunming mice (the experimental animal centre of the Second Affiliated Hospital of Harbin Medical University). The hearts were incubated with the mixture of trypsin and PBS 12 h at 4°C. Then, the heart tissue was digested with collagenaseII. Next, we collected the cell suspension and centrifuged at 1500 rpm for 7 min. Cells were re‐suspended in Dulbecco's Modified Eagle Medium (DMEM) with 10% fetal bovine serum and 1% penicillin‒streptomycin for 90 min. Suspended cardiomyocytes were collected and incubated on.1% gelatin‐coated plates with complete medium at 37°C in 5% CO_2_ for 48 h, then treated with various agents.

### 5.7 Wheat germ agglutinin dyeing

Remove frozen slices in ‒80°C refrigerator, using acetone fixation for 30 min. After PBS rinsing for three times, WGA with Alexa Fluor 594 diluted to 5 µmol/L was incubated at room temperature for 15 min.

### 5.8 Histological analysis

Cardiac tissue was fixed with 4% paraformaldehyde and embedded in paraffin. The 5 µm sections were stained with HE according to standard protocols. The cytoplasm is red and the nucleus is bluish brown.

### 5.9 Transmission electron microscopy

Post‐treatment, cardiomyocytes or 1 mm heart tissue were fixed in 2.5% glutaraldehyde (Paesel‐Lorei) in Hank's modified salt solution, post‐fixed in 1% OSO_4_, dehydrated in alcohol and embedded in Araldite (Serva). Ultrathin sections were prepared with an ultramicrotome (Leica), mounted on grids, and contrasted with lead citrate and uranyl acetate. A histologist used an electron microscope (H‐7650 Hitachi) to analyse and document ultrastructural images.

### 5.10 RNA immunoprecipitation

The Magna RIP RBP immunoprecipitation kit (Millipore) was performed for RIP experiments according to the manufacturer's instructions. Briefly, after lysing cardiac tissue samples with RIP lysis buffer, RNA‒protein complexes were captured using anti‐Zfp36, anti‐Ythdc2 and anti‐immunoglobulin G (IgG) antibodies, then precipitated with protein A/G magnetic beads. The protein was digested, and RNA was purified and reverse transcribed into cDNA. Ythdc2 and SLC7A11 mRNA levels were measured via quantitative PCR.

### 5.11 RNA‐pull down

The pull‐down assay was performed by RNA Antisense Purification Kit (BersinBio, Catalogue Bes5103). Neonatal ventricular myocytes were washed in PBS, cells were lysed by Lysis Buffer (BersinBio, Catalogue Bes5103), and used to perform pull‐down assays according to the manufacturer's instructions. RNA complexes were isolated by incubating cell lysates with biotin‐labelled Ythdc2/SLC7A11 mRNA probes and streptavidin beads. After washing the beads, protein sample buffer was added, heated and Zfp36/Ythdc2 were detected through western blotting using 10% sodium dodecyl sulphate‒polyacrylamide gel electrophoresis (SDS‒PAGE).

### 5.12 Dual luciferase reporter assays

The 3′UTRs of Ythdc2 and ACTB were cloned into dual luciferase reporter plasmids. HEK‐293T cells (2 × 10^4^ cells/well) were seeded in 24‐well plates and transfected with Ythdc2 or ACTB 3′UTR and Zfp36 or a control using Lipo2000 (Invitrogen). After 48 h, cells were washed with PBS, lysed with 100 µL passive lysis buffer, and shaken for 15 min. Next, luciferase activity was detected using dual luciferase reporter assay system (Promega). Renilla Luciferase Control Reporter Vectors (Promega) was used as internal control.

### 5.13 Immunofluorescence

Cardiomyocytes were fixed with paraformaldehyde, permeabilised with Triton X‐100 and blocked with goat serum. They were incubated overnight with anti‐sarcomeric alpha actinin antibody (EA‐53, GeneTex), followed by Dylight 594 goat anti‐mouse antibody for 1 h, and stained with 4',6‐diamidino‐2‐phenylindole (DAPI). Immunofluorescence was observed under a fluorescence microscope, and cell surface area was analysed using Image‐Pro Plus software.

### 5.14 Protein extraction and western blotting

Total protein was extracted from tissue and cell samples by adding RIPA lysis buffer (Beyotime), a protease inhibitor and phosphatase inhibitors (Sigma‒Aldrich). Proteins were separated by SDS‒PAGE and transferred to nitrocellulose membranes (Pall Corporation). Skim milk was blocked the nitrocellulose membranes for 2 h. Primary antibodies Zfp36, Ythdc2, SLC7A11, β‐MHC (Cat. no. 12737‐1‐AP, 27779‐1‐AP, 26864‐1‐AP, 22280‐1‐AP, Proteintech) and Gpx4 (Cat. no. A1933, ABclonal) were incubated overnight at 4°C. β‐Actin (Cat. no. 66009‐1‐Ig, Proteintech) was used as the internal control antibody. After incubation, the primary antibody was washed three times in PBST, then incubated secondary antibody for 60 min at room temperature. The ODYSSEY machine (LI‐COR) was used for scanning protein level content and qualitative analysis.

### 5.15 Quantitative real‐time PCR

Total RNA was extracted with TRIzol reagent (Invitrogen). Cells or tissues were fragmented with TRIzol reagent, and the total RNA was obtained by chloroform extraction and isopropanol precipitation. Reverse Transcription Kits (TransGen Biotech) were used to reverse transcribe the RNA into cDNA. To measure the mRNA expression levels of ANP, BNP, β‐MHC, Ptgs2, Zfp36, Ythdc2 and SLC7A11, real‐time PCR was carried out with SYBR Green (Roche).

Actin served as an internal control for mRNA quantification, with relative mRNA levels calculated using the 2^−ΔΔCT^ method based on threshold cycle (Ct) values.

The primer sequences used in this study are:

ANP forward: 5′‐CTCCGATAGATCTGCCCTCTTGAA‐3′
ANP reverse: 5′‐GGTACCGGAAGCTGTTGCAGCCTA‐3′
BNP forward: 5′‐GCTGCTGGAGCTGATAAGAGAA‐3′
BNP reverse: 5′‐GTTCTTTTGTAGGGCCTTGGTC‐3′
β‐MHC forward: 5′‐CCAGAAGCCTCGAAATGTC‐3′
β‐MHC reverse: 5′‐CTTTCTTTGCCTTGCCTTTGC‐3′
β‐Actin forward: 5′‐CTACCTCATGAAGATCCTGACC‐3′
β‐Actin reverse: 5′‐CACAGCTTCTCTTTGATGTCAC‐3′
SLC7A11 forward: 5′‐TGTGTTCGCTGTCTCCAGGTTATTC‐3′
SLC7A11 reverse: 5′‐GAGAAGAGCATCACCATCGTCAGAG‐3′
Ptgs2 forward: 5′‐AACACCTGAGCGGTTACCACTTC‐3′
Ptgs2 reverse: 5′‐AGAGGCAATGCGGTTCTGATACTG‐3′
Igf2bp2 forward: 5′‐AGAAGTTATCGTGCCTCGTGACC‐3′
Igf2bp2 reverse: 5′‐ATCTCTGCTCCTGCTGCTTCAC‐3′
Ythdc1 forward: 5′‐ACAGGGCAACAACACTGAGAATGAG‐3′
Ythdc1 reverse: 5′‐CTTCATCCTCTTCCTCCTCCTCCTC‐3′
Hnrnpa2b1 forward: 5′‐ATCCTGCAAGCAAAAGATCAAG‐3′
Hnrnpa2b1 reverse: 5′‐AACAAACAGCTTCTTCACAGTC‐3′
Ythdc2 forward: 5′‐CCTGTTACTGTCCTGGTGTTCTGTG‐3′
Ythdc2 reverse: 5′‐CTTCCATCTCACTGTCACTGCTGTC‐3′
Ythdf3 forward: 5′‐GCAGTTACGGCTATCCACCTAGTTC‐3′
Ythdf3 reverse: 5′‐AGTCCAGTCATGCCTTGCTCAATAC‐3′
Hnrnpc forward: 5′‐GGGTGACCTGCTGGATGATGAC‐3′
Hnrnpc reverse: 5′‐GCTGTCTCTGTCGTCTTCTCCTTC‐3′
Igf2bp3 forward: 5′‐CCACCAGAGGCTCAGTTCAAGG‐3′
Igf2bp3 reverse: 5′‐AGCAGCAAAGGACGGCACTC‐3′

### 5.16 MDA analysis

MDA in cellular and frozen myocardial tissues was measured using a kit (A003‐1‐2, Nanjing Jiancheng Bioengineering Institute), involving a reaction with thiobarbituric acid (TBA) to form a pink MDA‒TBA conjugate, detected at 532 nm (Infinite 200 PRO). Cellular MDA was expressed as nmol/mg protein.

### 5.17 GSH analysis

GSH in frozen myocardial tissue was measured using another kit (A005‐1‐2, Nanjing Jiancheng Bioengineering Institute), involving a reaction with DTNB, detected at 405 nm (Infinite 200 PRO), and expressed as nmol/mg protein.

### 5.18 ROS analysis

An ROS assay kit (S0033, Beyotime Biotech) was used to measure ROS generation levels in cardiomyocyte. The intracellular ROS indicator, 2',7'‐dichlorodihydrofluorescein diacetate (DCFH‐DA), was diluted to 10 mM in serum‐free medium and added to cells in a 96‐well plate for 30 min at 37°C. Fluorescence was measured using a Multi‐Function Measuring Instrument (Infinite 200 PRO) with excitation at 488 nm and emission at 525 nm.

### 5.19 Immunofluorescence‒fluorescence in situ hybridisation

IF‐FISH assay was conducted to locate Zfp36, Ythdc2 and SLC7A11 in cardiomyocytes. Cells were incubated with Zfp36 or Ythdc2 antibodies overnight at 4°C, followed by a Dylight 488 anti‐rabbit antibody for 1 h at room temperature. Cy3‐labelled mRNA probes for Ythdc2 and SLC7A11, designed by GenePharma, were hybridised overnight according to the manufacturer's instructions.

### 5.20 mRNA stability analysis

Transcriptional inhibitor actinomycin D (MCE, HY‐17 559) was used to inhibit RNA synthesis. After treatment with actinomycin D, CMs were harvested at 1, 2 and 4 h time points, and the RNA was then extracted. The remaining mRNA was detected through RT‐qPCR.

### 5.21 Construct crispr‐cas9 plasmid

Crispr‐cas9 plasmid carrying sgZfp36 was constructed by MiaoLingBio. According to the sgRNA sequence of the Zfp36 gene, annealing primers with sticky ends were designed respectively. BbsI were jointly connected to expression vector pAtU6‐26‐sgRNA‐35S‐EGFP‐Cas9 through single enzyme connection. According to the results of 1% agarose gel electrophoresis, the observed bands were consistent with the expected positions, and the size of the vector after single enzyme digestion. The sequencing results contain the target sequence of sgRNA, with a 100% match.

### 5.22 Statistical analysis

Experimental data underwent normality and homogeneity of variance tests. Results are shown as mean ± standard deviation from at least three independent experiments. Prior to conducting statistical analyses, all experimental data points were assessed for normality. A two‐tailed Student's *t*‐test was employed to compare data between two groups, while a one‐way analysis of variance was utilised for analysing and comparing data across multiple groups. For comparisons involving groups with non‐normally distributed data, non‐parametric methods were applied, specifically the Mann–Whitney *U*‐test for comparisons between one or two groups, and the Kruskal–Wallis test followed by Dunn's post hoc test for comparisons involving more than two groups. A *p*‐value of less than.05 indicated statistical significance.

## AUTHOR CONTRIBUTIONS

Mingyu Zhang supported the experimental design, conceptualized the study, supervised the project, and critically revised the manuscript for intellectual content. Xiaoxiang Guan designed and performed the experiments, analyzed data, and drafted the manuscript. Zheng Dong, Chenxu Yang, Chao Xiong, Wenzheng Cheng, Aijing Shang, Yaru Liu, Xiaofei Guo, Bowen Zhang, Bo Zhang, Saidi Jin, Wenyi Qi collected and summarised data. Berezhnova Tatjana Alexandrovna contributed to the manuscript revision. Yuan Jiang, Zhimin Du, Chaoqian Xu conceptualized the study, supervised the project, and critically revised the manuscript for intellectual content. All authors reviewed and approved the final manuscript.

## CONFLICT OF INTEREST STATEMENT

The authors declare they have no conflicts of interest.

## ETHICS STATEMENT

All animal handling protocols were approved by the Animal Care and Use Committee of Harbin Medical University (IRB5017622). All animal procedures conform to the guidelines from Directive 2010/63/EU of the European Parliament on the protection of animals used for scientific purposes or the NIH Guide for the Care and Use of Laboratory Animals.

## Supporting information



Supporting Information

## Data Availability

The data that support the findings of this study are available from the corresponding author upon reasonable request.

## References

[1] Li C , Li S , Zhang G , et al. Ire1α mediates the hypertrophic growth of cardiomyocytes through facilitating the formation of initiation complex to promote the translation of top‐motif transcripts. Circulation. 2024;150(13):1010‐1029. 38836349 10.1161/CIRCULATIONAHA.123.067606 PMC11427172

[2] Martin TG , Juarros MA , Leinwand LA . Regression of cardiac hypertrophy in health and disease: mechanisms and therapeutic potential. Nat Rev Cardiol. 2023;20(5):347‐363. 36596855 10.1038/s41569-022-00806-6 PMC10121965

[3] Nakamura M , Sadoshima J . Mechanisms of physiological and pathological cardiac hypertrophy. Nat Rev Cardiol. 2018;15(7):387‐407. 29674714 10.1038/s41569-018-0007-y

[4] Liu D , Qin H , Gao Y , Sun M , Wang M . Cardiovascular disease: mitochondrial dynamics and mitophagy crosstalk mechanisms with novel programmed cell death and macrophage polarisation. Pharmacol Res. 2024;206:107258. 38909638 10.1016/j.phrs.2024.107258

[5] Li L , Gao P , Tang X , et al. Cb1r‐stabilized Nlrp3 inflammasome drives antipsychotics cardiotoxicity. Signal Transduct Target Ther. 2022;7(1):190. 35739093 10.1038/s41392-022-01018-7 PMC9225989

[6] Gao P , Cao M , Jiang X , et al. Cannabinoid receptor 2‐centric molecular feedback loop drives necroptosis in diabetic heart injuries. Circulation. 2023;147(2):158‐174. 36448459 10.1161/CIRCULATIONAHA.122.059304

[7] Yu H , Gan D , Luo Z , et al. α‐Ketoglutarate improves cardiac insufficiency through NAD+‐SIRT1 signaling‐mediated mitophagy and ferroptosis in pressure overload‐induced mice. Mol Med. 2024;30(1):15. 38254035 10.1186/s10020-024-00783-1 PMC10804789

[8] Dixon SJ , Olzmann JA . The cell biology of ferroptosis. Nat Rev Mol Cell Biol. 2024;25(6):424‐442. 38366038 10.1038/s41580-024-00703-5 PMC12187608

[9] Fang X , Ardehali H , Min J , Wang F . The molecular and metabolic landscape of iron and ferroptosis in cardiovascular disease. Nat Rev Cardiol. 2023;20(1):7‐23. 35788564 10.1038/s41569-022-00735-4 PMC9252571

[10] Bi X , Wu X , Chen J , et al. Characterization of ferroptosis‐triggered pyroptotic signaling in heart failure. Signal Transduct Target Ther. 2024;9(1):257. 39327446 10.1038/s41392-024-01962-6 PMC11427671

[11] Li T , Sun M , Sun Q , et al. Pm(2.5)‐induced iron homeostasis imbalance triggers cardiac hypertrophy through ferroptosis in a selective autophagy crosstalk manner. Redox Biol. 2024;72:103158. 38631121 10.1016/j.redox.2024.103158 PMC11033202

[12] Zhang K , Tian XM , Li W , Hao LY . Ferroptosis in cardiac hypertrophy and heart failure. Biomed Pharmacother. 2023;168:115765. 37879210 10.1016/j.biopha.2023.115765

[13] Diaz‐Muñoz MD , Osma‐Garcia IC . The RNA regulatory programs that govern lymphocyte development and function. Wiley Interdiscip Rev RNA. 2022;13(1):e1683. 34327847 10.1002/wrna.1683

[14] Snyder BL , Blackshear PJ . Clinical implications of tristetraprolin (Ttp) modulation in the treatment of inflammatory diseases. Pharmacol Ther. 2022;239:108198. 35525391 10.1016/j.pharmthera.2022.108198 PMC9636069

[15] Yadav S , El Hamra R , Alturki NA , et al. Regulation of Zfp36 by Isgf3 and Mk2 restricts the expression of inflammatory cytokines during necroptosis stimulation. Cell Death Dis. 2024;15(8):574. 39117638 10.1038/s41419-024-06964-4 PMC11310327

[16] Li X , Hu Y , Wu Y , Yang Z , Liu Y , Liu H . Exosomal Let‐7a‐5p derived from human umbilical cord mesenchymal stem cells alleviates coxsackievirus B3‐induced cardiomyocyte ferroptosis via the Smad2/Zfp36 signal axis. J Zhejiang Univ Sci B. 2024;25(5):422‐437. 38725341 10.1631/jzus.B2300077 PMC11087186

[17] Zhang M , Zhang X , Niu J , Hua C , Liu P , Zhong G . Integrated analysis of single‐cell RNA sequencing and bulk RNA data reveals gene regulatory networks and targets in dilated cardiomyopathy. Sci Rep. 2024;14(1):13942. 38886541 10.1038/s41598-024-64693-2 PMC11183045

[18] Cui X , Wang Y , Lu H , et al. Zfp36 regulates vascular smooth muscle contraction and maintains blood pressure. Adv Sci. 2025;12(3):e2408811. 10.1002/advs.202408811 PMC11744710 39589932

[19] Liu J , Cao X . RBP‒RNA interactions in the control of autoimmunity and autoinflammation. Cell Res. 2023;33(2):97‐115. 36599968 10.1038/s41422-022-00752-5 PMC9892603

[20] Wang M , Li Y , Xiao Y , et al. Nicotine‐mediated Otud3 downregulation inhibits Vegf‐C Mrna decay to promote lymphatic metastasis of human esophageal cancer. Nat Commun. 2021;12(1):7006. 34853315 10.1038/s41467-021-27348-8 PMC8636640

[21] Ma L , Chen T , Zhang X , et al. The M(6)a reader Ythdc2 inhibits lung adenocarcinoma tumorigenesis by suppressing Slc7a11‐dependent antioxidant function. Redox Biol. 2021;38:101801. 33232910 10.1016/j.redox.2020.101801 PMC7691619

[22] Koppula P , Zhang Y , Zhuang L , Gan B . Amino acid transporter Slc7a11/Xct at the crossroads of regulating redox homeostasis and nutrient dependency of cancer. Cancer Commun. 2018;38(1):12. 10.1186/s40880-018-0288-x PMC5993148 29764521

[23] Chen K , Wang S , Sun QW , Zhang B , Ullah M , Sun Z . Klotho deficiency causes heart aging via impairing the Nrf2‐Gr pathway. Circ Res. 2021;128(4):492‐507. 33334122 10.1161/CIRCRESAHA.120.317348 PMC8782577

[24] Peng F , Liao M , Jin W , et al. 2‐Apqc, a small‐molecule activator of sirtuin‐3 (Sirt3), alleviates myocardial hypertrophy and fibrosis by regulating mitochondrial homeostasis. Signal Transduct Target Ther. 2024;9(1):133. 38744811 10.1038/s41392-024-01816-1 PMC11094072

[25] Maremonti F , Tonnus W , Gavali S , et al. Ferroptosis‐based advanced therapies as treatment approaches for metabolic and cardiovascular diseases. Cell Death Differ. 2024;31(9):1104‐1112. 39068204 10.1038/s41418-024-01350-1 PMC11369293

[26] Zhang W , Lu J , Wang Y , et al. Canagliflozin attenuates lipotoxicity in cardiomyocytes by inhibiting inflammation and ferroptosis through activating Ampk pathway. Int J Mol Sci. 2023;24(1):858. 36614295 10.3390/ijms24010858 PMC9821072

[27] Yuan J , Yin C , Peng H , et al. Ndrg1 regulates iron metabolism and inhibits pathologic cardiac hypertrophy. Can J Cardiol. 2024;41(2):224‐240. 39427843 10.1016/j.cjca.2024.10.011

[28] Yoshida M , Minagawa S , Araya J , et al. Involvement of cigarette smoke‐induced epithelial cell ferroptosis in COPD pathogenesis. Nat Commun. 2019;10(1):3145. 31316058 10.1038/s41467-019-10991-7 PMC6637122

[29] Xu W , Billon C , Li H , et al. Novel Pan‐Err agonists ameliorate heart failure through enhancing cardiac fatty acid metabolism and mitochondrial function. Circulation. 2024;149(3):227‐250. 37961903 10.1161/CIRCULATIONAHA.123.066542 PMC10842599

[30] Alsereidi FR , Khashim Z , Marzook H , et al. Dapagliflozin mitigates cellular stress and inflammation through Pi3k/Akt pathway modulation in cardiomyocytes, aortic endothelial cells, and stem cell‐derived Β cells. Cardiovasc Diabetol. 2024;23(1):388. 39472869 10.1186/s12933-024-02481-y PMC11520772

[31] Qi Y , Xu H , Li X , et al. Silica nanoparticles induce cardiac injury and dysfunction via Ros/Ca(2+)/Camkii signaling. Sci Total Environ. 2022;837:155733. 35526619 10.1016/j.scitotenv.2022.155733

[32] Qiu H , Sun Y , Wang X , et al. Lamin A/C deficiency‐mediated Ros elevation contributes to pathogenic phenotypes of dilated cardiomyopathy in IPSC model. Nat Commun. 2024;15(1):7000. 39143095 10.1038/s41467-024-51318-5 PMC11324749

[33] Newton K , Strasser A , Kayagaki N , Dixit VM . Cell death. Cell. 2024;187(2):235‐256. 38242081 10.1016/j.cell.2023.11.044

[34] Shen M , Cao S , Long X , et al. Dnajc12 causes breast cancer chemotherapy resistance by repressing doxorubicin‐induced ferroptosis and apoptosis via activation of Akt. Redox Biol. 2024;70:103035. 38306757 10.1016/j.redox.2024.103035 PMC10847378

[35] Yuan J , Ofengeim D . A guide to cell death pathways. Nat Rev Mol Cell Biol. 2024;25(5):379‐395. 38110635 10.1038/s41580-023-00689-6

[36] Baumgarten G , Knuefermann P , Kalra D , et al. Load‐dependent and ‐independent regulation of proinflammatory cytokine and cytokine receptor gene expression in the adult mammalian heart. Circulation. 2002;105(18):2192‐2197. 11994254 10.1161/01.cir.0000015608.37608.18

[37] Zhao J , Yang S , Lv C , Liu Y . Cancer‐associated fibroblasts suppressed ferroptosis in glioblastoma via upregulating Lncrna Dleu1. Am J Physiol Cell Physiol. 2023;324(5):C1039‐C1052. 36878845 10.1152/ajpcell.00454.2022

[38] Cicchetto AC , Jacobson EC , Sunshine H , et al. Zfp36‐mediated Mrna decay regulates metabolism. Cell Rep. 2023;42(5):112411. 37086408 10.1016/j.celrep.2023.112411 PMC10332406

[39] Wang W , Zhang TN , Yang N , et al. Transcriptome‐wide identification of altered RNA M(6)a profiles in cardiac tissue of rats with LPS‐induced myocardial injury. Front Immunol. 2023;14:1122317. 37275860 10.3389/fimmu.2023.1122317 PMC10237353

[40] Zhang X , Zheng C , Gao Z , et al. Slc7a11/Xct prevents cardiac hypertrophy by inhibiting ferroptosis. Cardiovasc Drugs Ther. 2022;36(3):437‐447. 34259984 10.1007/s10557-021-07220-z

