# Supplementary material for: Targeting Zfp36 to combat cardiac hypertrophy: Insights into ferroptosis pathways
Source: Clin Transl Med. 2025 Feb 25;15(3):e70247. doi: 10.1002/ctm2.70247 (PMC11859123; doi:10.1002/ctm2.70247)
Supplement: Supplementary file 1 — Supporting Information [file CTM2-15-e70247-s001.docx]

Supplements


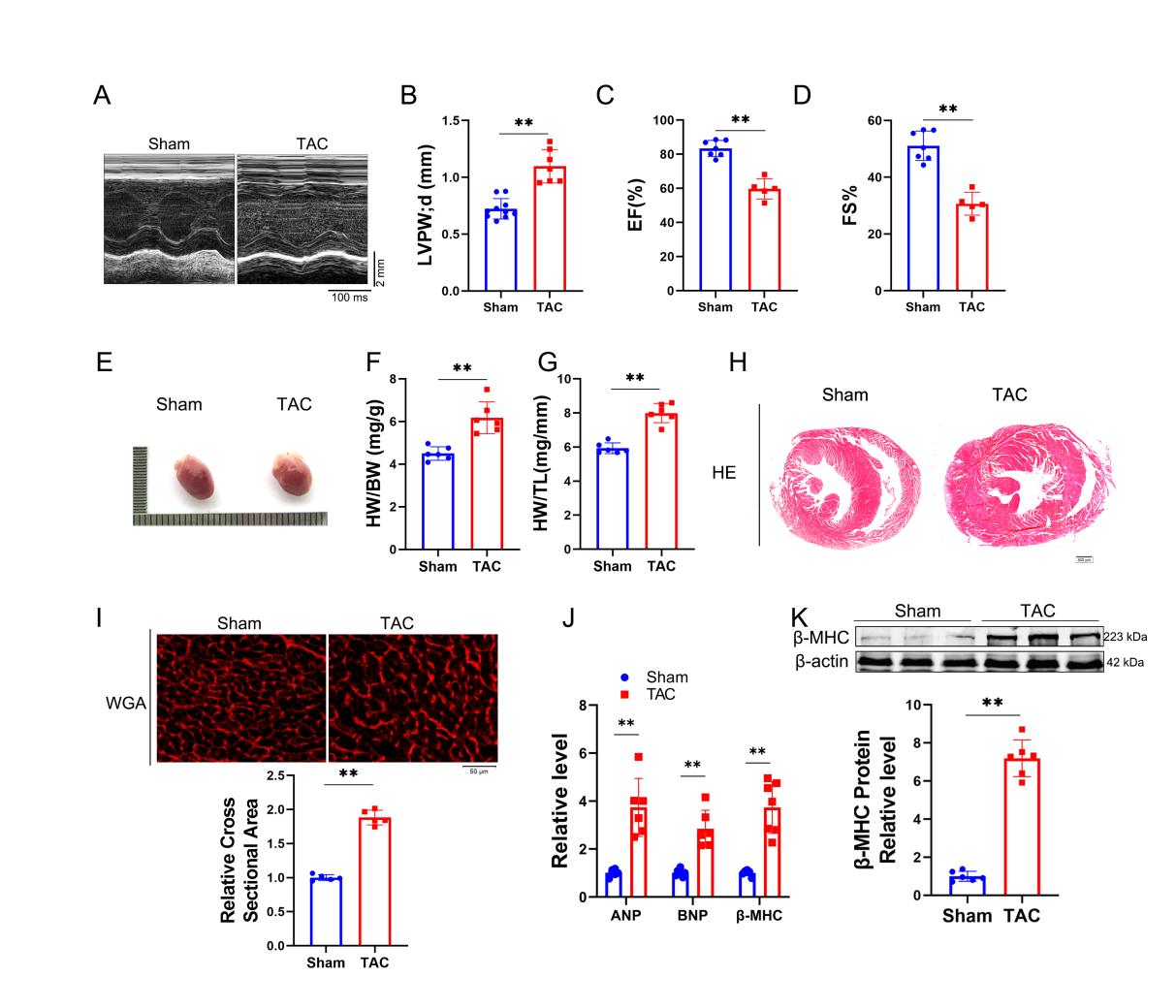


**Supplement 1. Cardiac hypertrophy induced by TAC in mice.**

(A) Representative echocardiography recordings in mice. (B) The left ventricular posterior wall thickness dimensions during diastole (LVPW; d) in mice, n=7-10. (C, D) Quantification of EF% and FS% among mice, n=5-7. (E) Gross appearance of whole heart. (F) Heart weight (HW) to body weight (BW) ratio, n=6. (G) Heart weight (HW) to tibia length ratio (TL), n=6. (H) Representative images of heart sections stained with HE. Scale bars=500μm. (I) The representative photographs of heart sections stained with WGA and Quantification of WGA, Scale bars=50μm, n=5. (J) qRT-PCR analyzed the mRNA expression levels of ANP, BNP and β-MHC in Sham and TAC group mice hearts, n=6-7. (K) Western Blot results shown the protein expression level of β-MHC in mice hearts, n=6. Results presented as mean ± SD. Statistical analysis was performed using a two-way t-tests followed by Bonferroni significant difference post hoc test. **P<0.01.


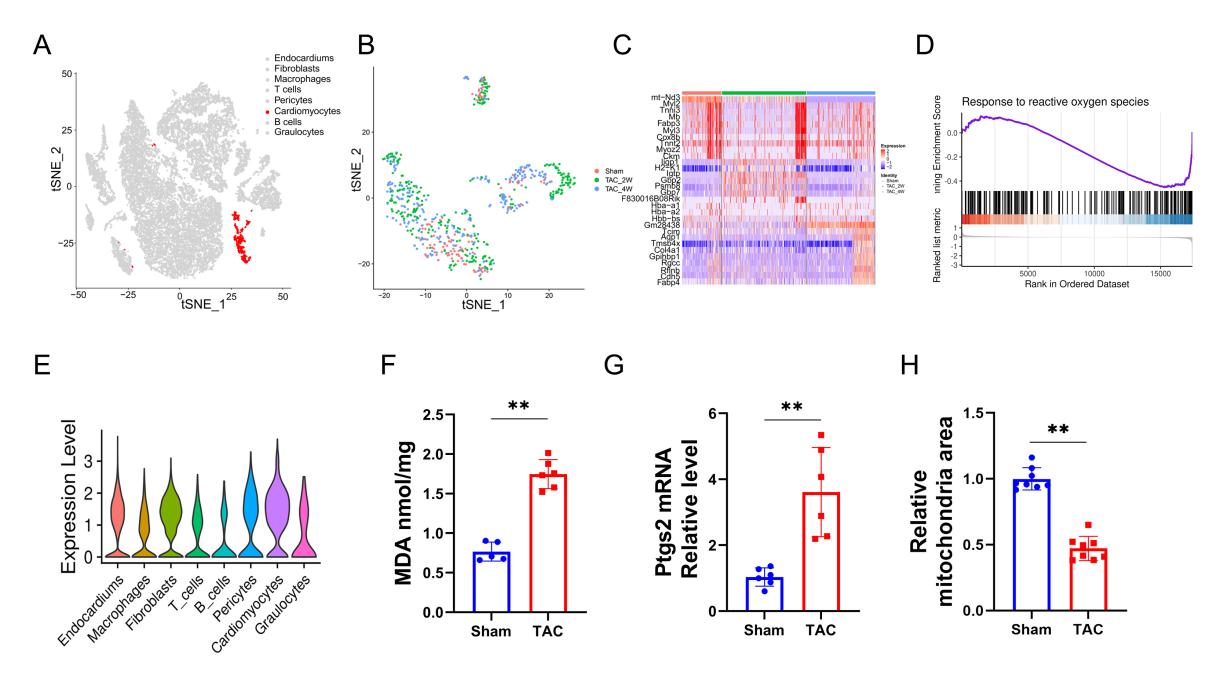


**Supplement 2. Genes related to ferroptosis were up-regulated in cardiac hypertrophy.**

(A) FIt-SNE plot with cells colored according to cardiomyocyte marker genes transcript abundance. (B) t-SNE projection of cardiomyocytes analyzed by scRNA-seq. (C) Transcriptomic profiles of cardiomyocytes were drastically altered by post-TAC operation. (D) GSEA showing that the genes related to response to ROS were up-regulated in TAC mice. (E) High expression of Gpx4 in cardiomyocytes. (F) Detection of MDA for lipid peroxidation level in Sham and TAC mice, n=5-6. (G) qRT-PCR analyzed the mRNA expression levels of Ptgs2 in mice hearts, n=6. (H) Quantification of mitochondriea area in transmission electron microscopymice image among mice hearts. Statistical analysis was performed with Student’s t-test. Results presented as mean ± SD. **P<0.01.

**
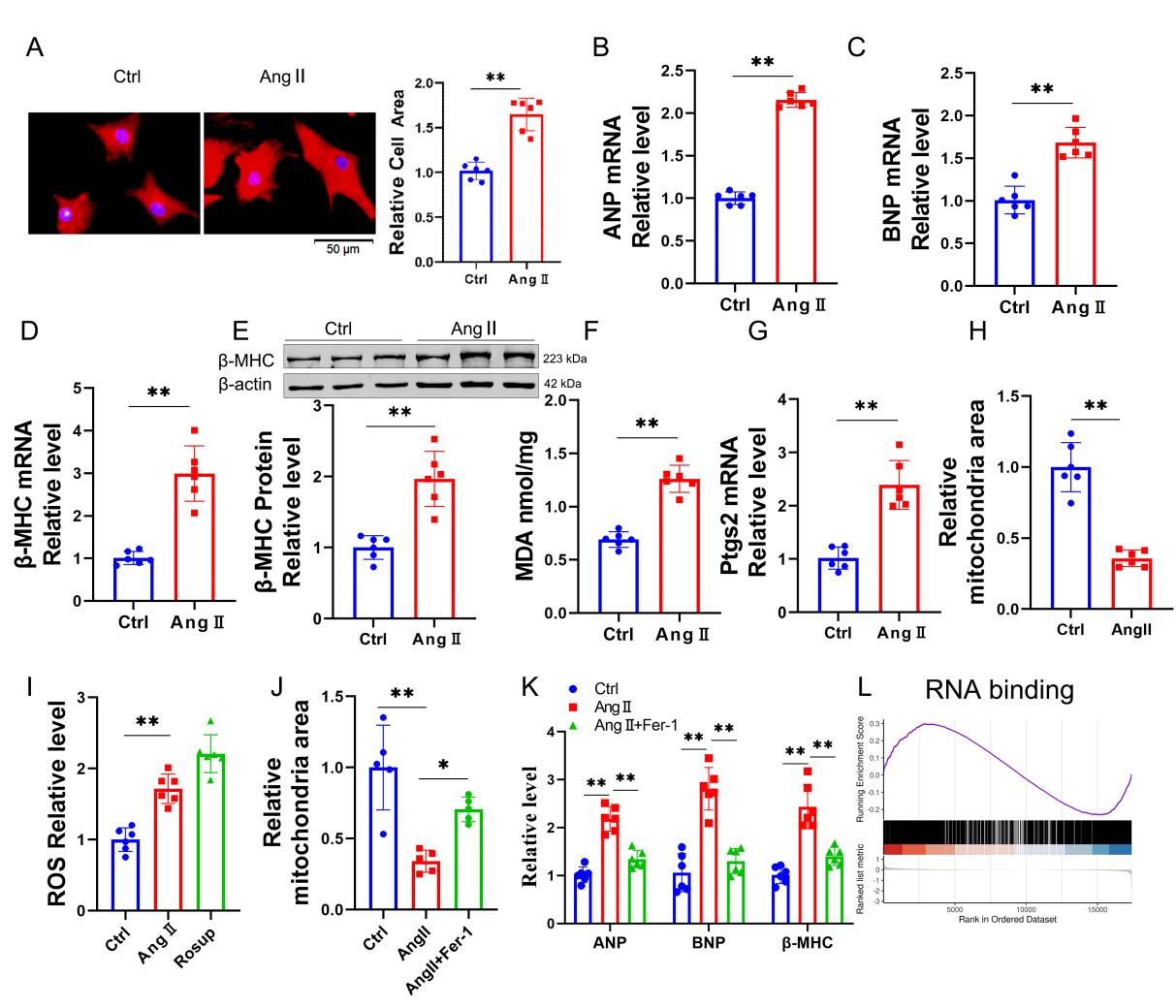
**

**Supplement 3. Cardiomyocytes hypertrophy induced by AngII.**

(A)The representative photographs and averaged cell area of cardiomyocytes identified with α-actinin antibody (red), and nuclei were stained with DAPI (blue), Scale bars, 50μm, n=6. (B-D) qRT-PCR analyzed the mRNA expression levels of ANP, BNP and β-MHC in cardiomyocytes, n=6. (E) Western Blot results shown the protein expression level of β-MHC, n=6. (F) Detection of MDA level in cardiomyocytes, n=6. (G) qRT-PCR analyzed the mRNA expression level of Ptgs2, n=6. (H, J) Quantification of mitochondriea area in transmission electron microscopymice image among cardiomyocytes. (I) DCFH-DA probe staining for the ROS levels of cardiomyocytes after treating with AngII, n=6. (K) qRT-PCR analyzed the mRNA expression levels of ANP, BNP and β-MHC in cardiomyocytes, n=6. (L) GSEA showing that the genes related to RNA-binding in TAC mice. Statistical analysis was performed with Student’s t-test or one-way ANOVA. Results presented as mean ± SD. *P<0.05,**P<0.01.


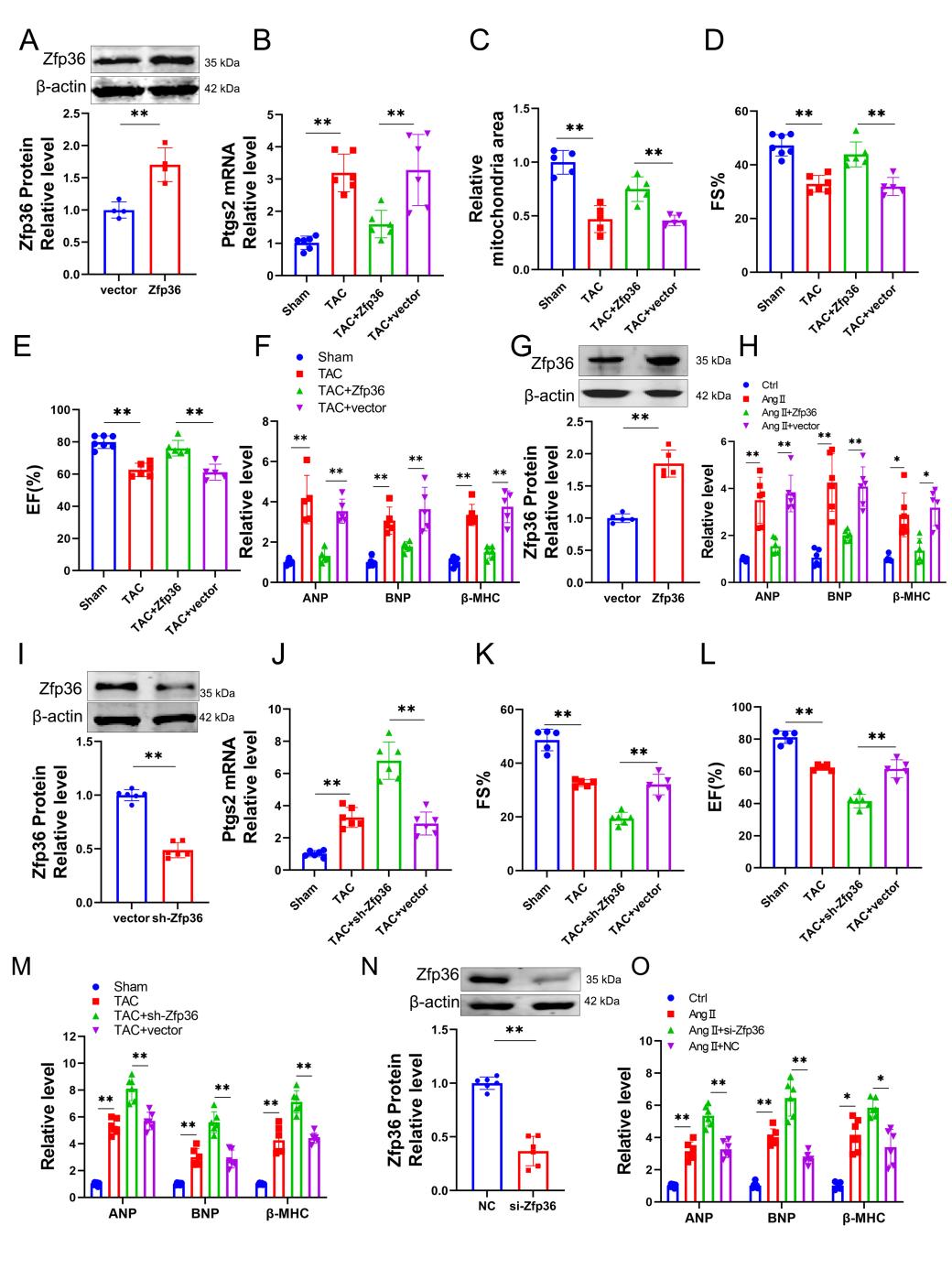


**Supplement 4. Zfp36 attenuated ferroptosis and cardiac hypertrophy.**

(A, G, I, N) Overexpression or knock-down efficacy of Zfp36 was validated through Western Blot, n=4-6. (B, J) qRT-PCR analyzed the mRNA expression levels of Ptgs2 in mice hearts, n=6. (C) Quantification of mitochondriea area in transmission electron microscopymice image among mice hearts. (D, E, K, L) Quantification of EF% and FS% in mice, n=5-6. (F, H, M, O) qRT-PCR analyzed the mRNA expression levels of ANP, BNP and β-MHC, n=5-6. Statistical analysis was performed with Student’s t-test or one-way ANOVA. Results presented as mean ± SD. *P<0.05, **P<0.01.


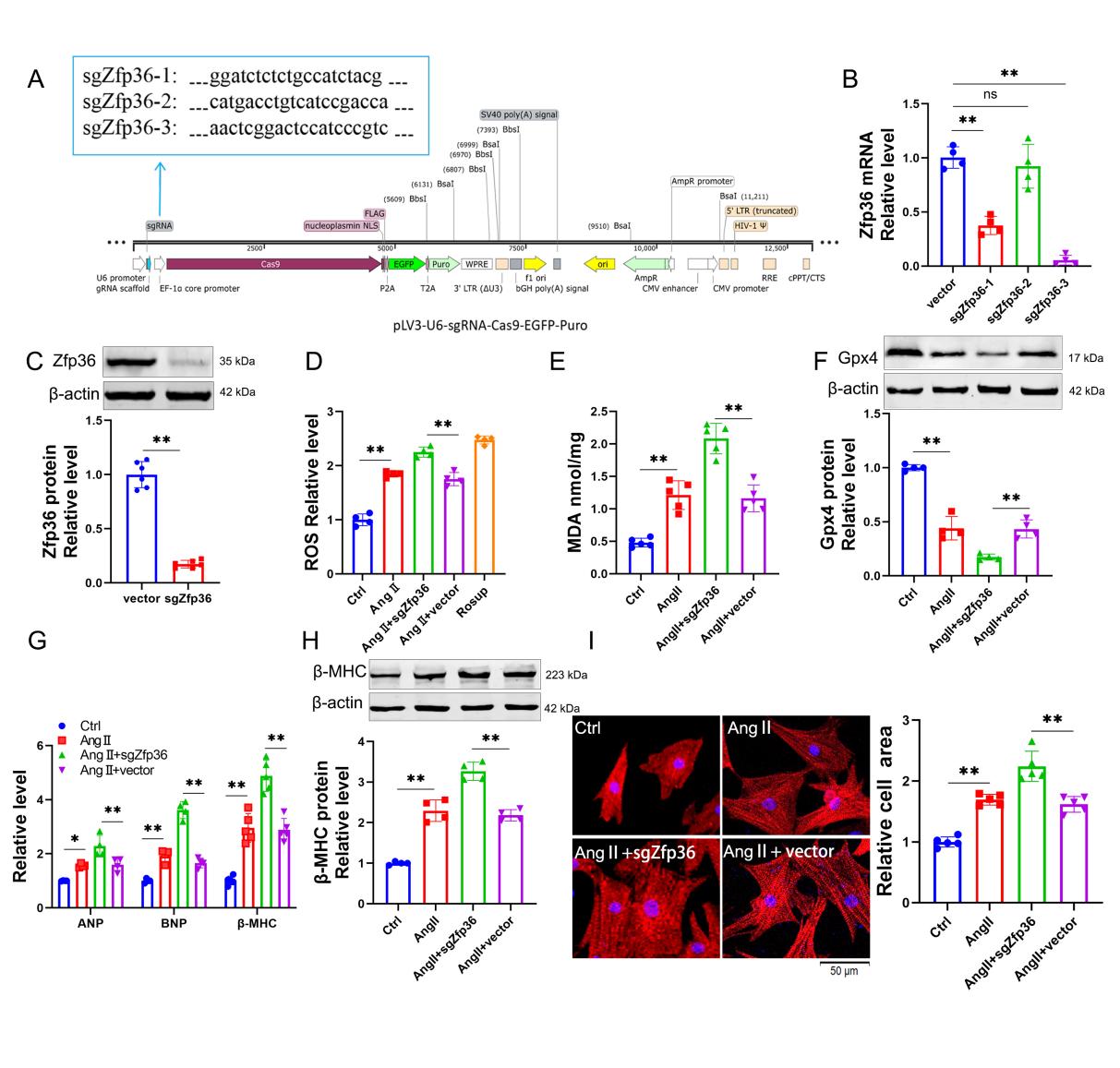


**Supplement 5: sgZfp36 promotes ferroptosis and aggravates cardiomyocyte hypertrophy.** (A) The map of crispr-cas9 vector and sgRNA sequence targeting Zfp36. (B) qRT-PCR analyzed the mRNA expression levels of Zfp36 in cardiomyocytes, n=4. (C) Western Blot results showed the expression level of Zfp36, n=6. (D) DCFH-DA probe determined the levels of ROS in cardiomyocytes, n=4. (E) Detection of MDA for lipid peroxidation level, n=5. (F) Western blot analyzed the expression levels of Gpx4, n=4. (G) qRT-PCR analyzed the mRNA expression levels of ANP, BNP and β-MHC in cardiomyocytes, n=4-5. (H) Western Blot results shown the protein expression level of β-MHC, n=4. (I) The representative photographs of cardiomyocytes identified with α-actinin antibody (red), and nuclei was stained with DAPI (blue), Scale bars=50μm, n=5-6. Statistical analysis was performed with Student’s t-test or one-way ANOVA. Results presented as mean ± SD. *P<0.05, **P<0.01.


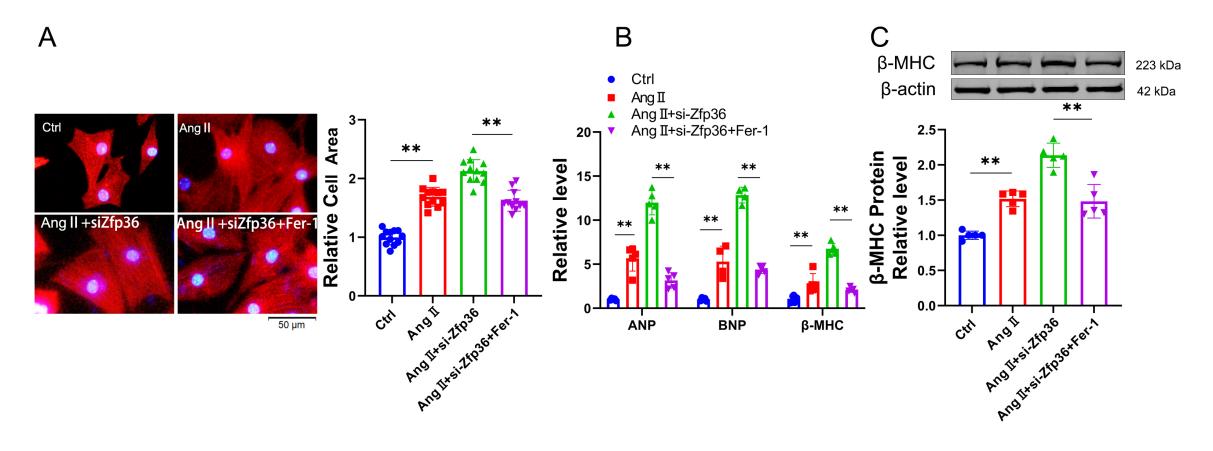


**Supplement 6.** **Zfp36 modulates cardiac hypertrophy through ferroptosis.**

(A) The representative photographs of cardiomyocytes identified with α-actinin antibody (red), and nuclei was stained with DAPI (blue), Scale bars, 50μm and the averaged data of cell area stained by α-actinin, n=11-12. (B) qRT-PCR analyzed the mRNA expression levels of ANP, BNP and β-MHC in cardiomyocytes, n=4-5. (C) Western Blot shown the protein expression level of β-MHC, n=5. Statistical analysis was performed with one-way ANOVA. Results presented as mean ± SD. **P<0.01.


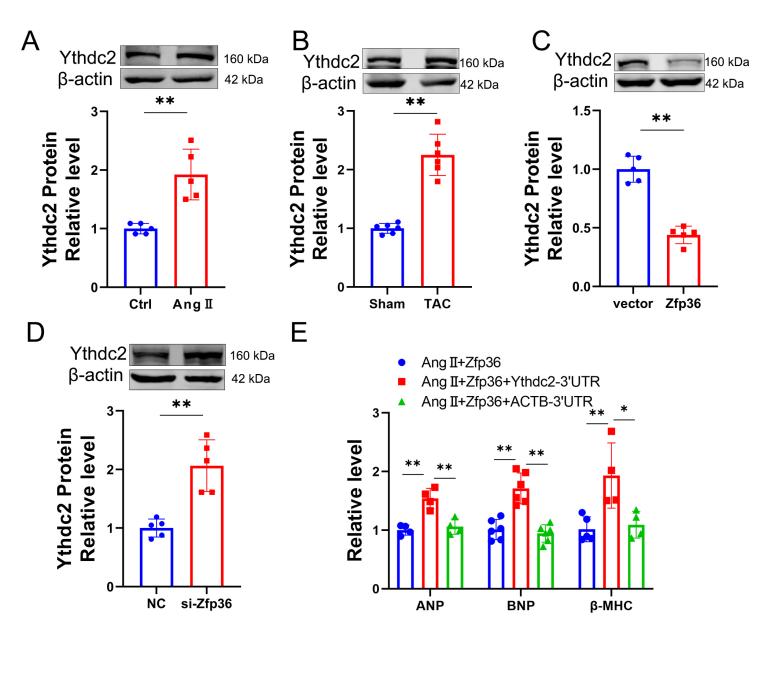


**Supplement 7. Ythdc2 is a direct target of Zfp36.**

(A-D) Western Blot shown the protein expression level of Ythdc2, n=5-6. (E) qRT-PCR analyzed the mRNA expression levels of ANP, BNP and β-MHC, n=4-6. Statistical analysis was performed with Student’s t-test or one-way ANOVA. Results presented as mean ± SD. *P<0.05, **P<0.01.


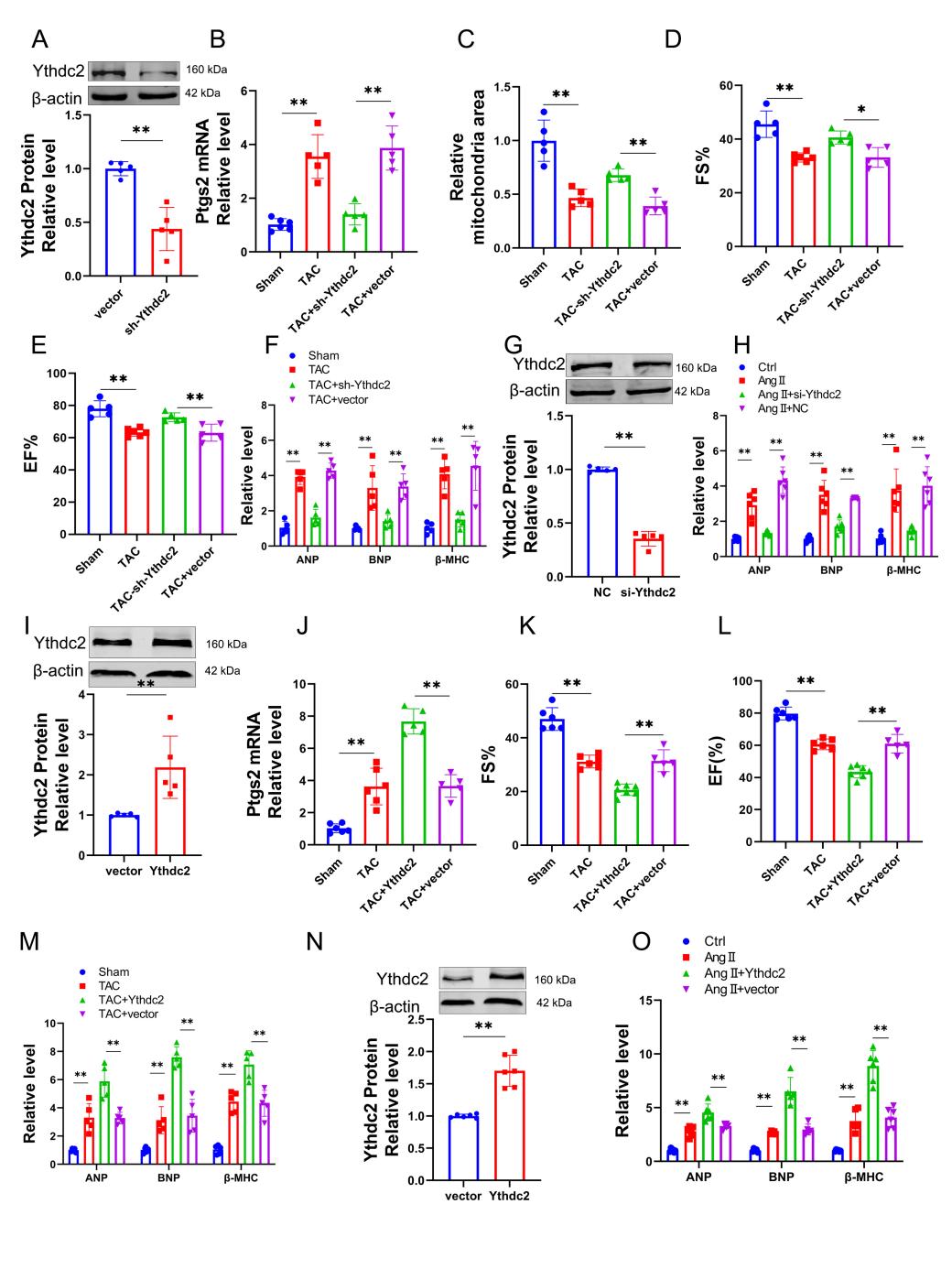


Supplement 8. Ythdc2 exacerbates ferroptosis in hypertrophy. (A, G, I, N) Overexpression or knock-down efficacy of Ythdc2 was validated through Western Blot, n=5-6. (B, J) qRT-PCR analyzed analyzed the mRNA expression levels of Ptgs2 in mice hearts, n=5-6. (C) Quantification of mitochondriea area in transmission electron microscopymice image among mice hearts, n=5. (D, E, K, L) Quantification of EF% and FS% in mice, n=5-6. (F, H, M, O) qRT-PCR analyzed the mRNA expression levels of ANP, BNP and β-MHC, n=5-6. Statistical analysis was performed with Student’s t-test or one-way ANOVA. Results presented as mean ± SD. *P<0.05, **P<0.01.


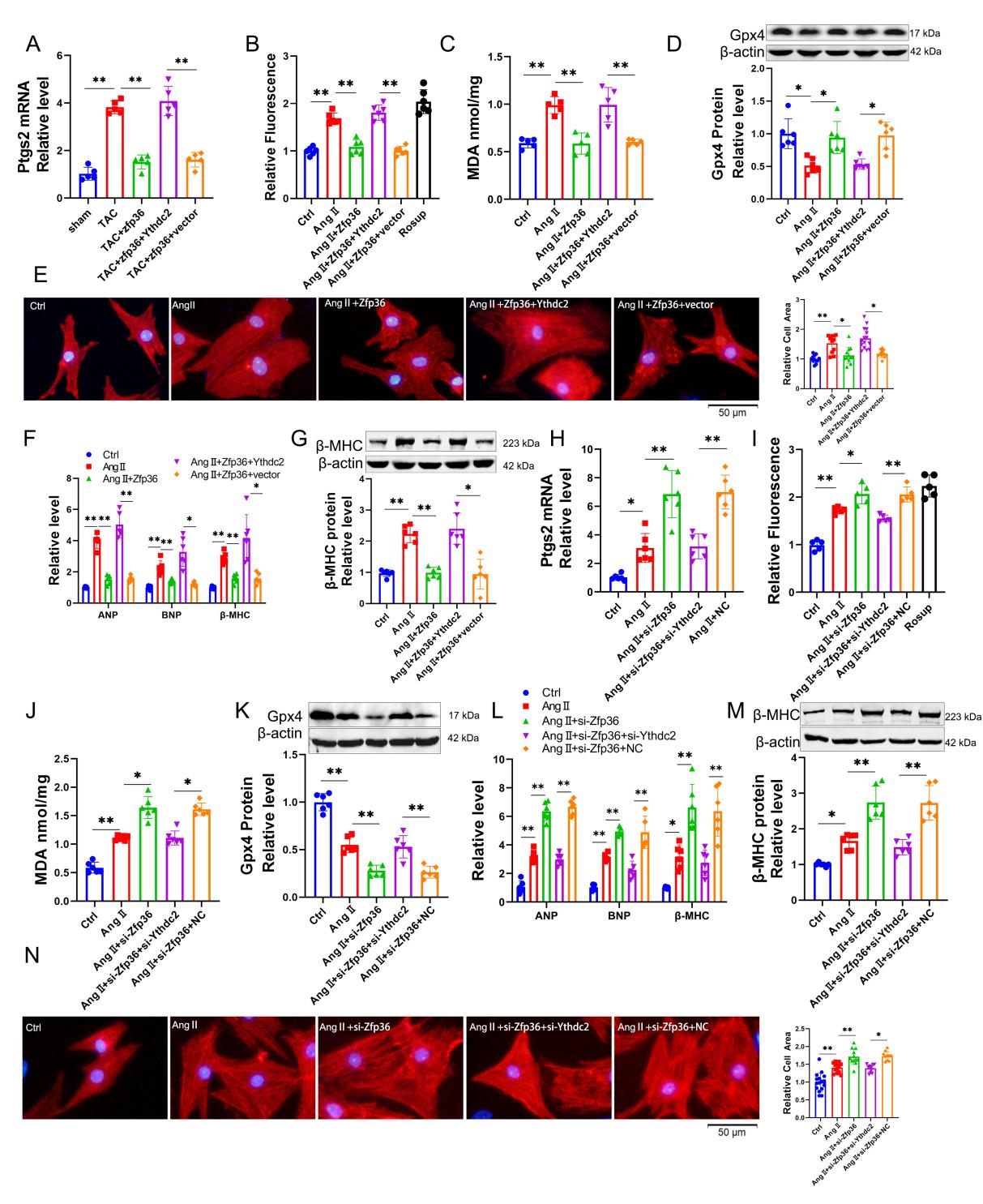


**Supplement 9. Regulated of Ythdc2 expression by Zfp36 mediates the ferroptosis and hypertrophy phenotypes in cardiomyocytes.**

(A, H) qRT-PCR analyzed the mRNA expression levels of Ptgs2, n=5-6. (B, I) DCFH-DA probe staining for the ROS levels of cardiomyocytes, n=5-6. (C, J) Detection of MDA for lipid peroxidation level, n=5-6. (D, K) Western blot analyzed the expression levels of Gpx4, n=6. (E, N) The representative photographs of cardiomyocytes identified with α-actinin antibody (red), nuclei were stained with DAPI (blue), Scale bars, 50μm, and the averaged data of cell area stained by α-actinin, n=10-16. (F, L) qRT-PCR analyzed the mRNA expression levels of ANP, BNP and β-MHC in cardiomyocytes, n=5-6. (G, M) Western Blot results shown the protein expression level of β-MHC, n=6. Statistical analysis was performed with Student’s t-test or one-way ANOVA. Results presented as mean ± SD. *P<0.05, **P<0.01.
